# Cost-effectiveness analysis of serplulimab plus chemotherapy in the first-line treatment for PD-L1-positive esophageal squamous cell carcinoma in China

**DOI:** 10.3389/fimmu.2023.1172242

**Published:** 2023-05-04

**Authors:** Shixian Liu, Nana Jiang, Lei Dou, Shunping Li

**Affiliations:** ^1^ Centre for Health Management and Policy Research, School of Public Health, Cheeloo College of Medicine, Shandong University, Jinan, China; ^2^ NHC Key Laboratory of Health Economics and Policy Research (Shandong University), Jinan, China; ^3^ Center for Health Preference Research, Shandong University, Jinan, China; ^4^ Department of Maternal and Child Health, School of Public Health, Cheeloo College of Medicine, Shandong University, Jinan, China

**Keywords:** cost-effectiveness, esophageal squamous-cell carcinoma, serplulimab, first-line, immunotherapy

## Abstract

**Objective:**

The ASTRUM-007 trial (NCT03958890) demonstrated that serplulimab plus chemotherapy administered every 2-week significantly improved progression-free and overall survival in patients with previously untreated, programmed death-ligand 1 (PD-L1) positive advanced esophageal squamous-cell carcinoma (ESCC). This study was aimed to investigate the cost-effectiveness of serplulimab plus chemotherapy in the first-line treatment of PD-L1-positive advanced ESCC.

**Methods:**

A partitioned survival model with a 2-week cycle and a 10-year time horizon was constructed from the Chinese healthcare system perspective. The survival data, direct medical costs and utilities were derived from the ASTRUM-007 trial, YAOZHI database and published sources. Total costs, quality-adjusted life-years (QALYs) and incremental cost-effectiveness ratios (ICERs) were calculated. Scenario, one-way and probabilistic sensitivity analyses were performed to assess the uncertainty around model parameters.

**Results:**

Compared with chemotherapy, serplulimab plus chemotherapy provided additional 0.27 QALYs with an incremental cost of $33,460.86, which had an ICER of $124,483.07 per QALY. The subgroup analyses revealed that the ICERs of serplulimab plus chemotherapy were $134,637.42 and $105,589.71 in advanced ESCC patients with 1 ≤ CPS < 10 and CPS ≥ 10, respectively. The price of serplulimab, patient weight, utility values and discount rate were the most influential parameters on base-case results. At a willingness-to-pay threshold of three times per capita GDP ($40,587.59) in 2022, the probability of serplulimab plus chemotherapy being cost-effective was 0% compared with chemotherapy. When the price of serplulimab decreased by 70%, the probabilities of serplulimab plus chemotherapy being cost-effective were 81.42%, 67.74% and 96.75% in advanced ESCC patients with PD-L1-positive, PD-L1 1≤CPS<10 and CPS≥10, respectively.

**Conclusion:**

Serplulimab plus chemotherapy in the first-line treatment for PD-L1-positive advanced ESCC might not be cost-effective in China.

## Introduction

Esophageal cancer is the fifth most common malignancy and the fourth leading cause of cancer-related death in China (1, 2). Esophageal squamous cell carcinoma (ESCC) and esophageal adenocarcinoma represent the predominant histological subtype, with the former accounting for approximately 85% of cases (3). Fluoropyrimidine or paclitaxel plus cisplatin-based chemotherapy remains the standard first-line treatment for patients with advanced or metastatic ESCC, which generally carried an extremely poor prognosis with median overall survival (OS) of fewer than 1 year (4, 5). Therefore, there is an unmet need for revolutionary therapeutic strategies to improve survival rates in advanced ESCC patients.

The emergence of immune checkpoint inhibitors targeting programmed death 1 (PD-1) or programmed death-ligand 1 (PD-L1) has drastically altered the landscape of cancer treatment (6). Serplulimab (HLX10), a fully humanized immune-globulin G4 monoclonal antibody against the PD-1 receptor, showed clinical efficacy in multiple malignancies (7, 8). Serplulimab has been approved by the National Medical Products Administration in the treatment of microsatellite instability-high (MSI-H) solid tumors, non-squamous non-small cell lung cancer and extensive-stage small-cell lung cancer in China (9).

Recently, ASTRUM-007, a randomized, double-blind, phase III clinical trial conducted at 70 hospitals in China, assessed the efficacy and safety of serplulimab plus chemotherapy compared with mono-chemotherapy as the first-line treatment of advanced or metastatic ESCC patients with PD-L1 combined positive score (CPS) ≥ 1 (10). The results demonstrated that serplulimab plus chemotherapy significantly prolonged median progression-free survival (PFS) (5.8 months vs. 5.3 months; hazard ratio [HR]: 0.60, 95% confidence interval [CI]: 0.48-0.75) and OS (15.3 months vs. 11.8 months; HR: 0.68, 95% CI: 0.53-0.87) in comparison with placebo plus chemotherapy (10). Compared with PD-L1 CPS < 10 patients, advanced ESCC patients with PD-L1 CPS ≥ 10 achieved better median PFS (7.1 months vs. 5.7 months) and OS (18.6 months vs. 14.2 months) outcomes from serplulimab in combination with chemotherapy. In terms of safety, the incidences of grade 3 or higher treatment-related adverse events (AEs) were 53% and 48% for serplulimab plus chemotherapy and chemotherapy, respectively.

Despite the enthusiasm surrounding serplulimab with superior efficacy, its cost-effectiveness remains unclear but is imperative for health decision-making and clinical practice. Therefore, this study aimed to evaluate the cost-effectiveness of serplulimab plus chemotherapy as the first-line treatment for previously untreated, PD-L1-positive (CPS ≥ 1) advanced ESCC from the perspective of Chinese healthcare system. Such evidence might provide guidance for clinicians and support reimbursement policy to optimize health resource allocation.

## Methods

### Patients and treatment

This study was reported in accordance with the Consolidated Health Economic Evaluation Reporting Standards 2022 (CHEERS 2022) updated reporting guidelines (**Supplementary Table S1**) (11). In this institutional review board-exempt economic evaluation, targeted patients were aged 18-75 years with previously untreated, histologically confirmed, inoperable locally advanced or metastatic, PD-L1-positive (CPS ≥ 1) ESCC, with at least one measurable lesion based on central imaging in line with response evaluation criteria in solid tumors v1.1, adequate organ function, and Eastern Cooperative Oncology Group performance status 0-1 (10). Patients who had previously received PD-1 or PD-L1 inhibitors, had central nervous system metastases or presented with active infection or active autoimmune diseases were excluded (10).

Eligible patients received serplulimab (3 mg/kg) or placebo intravenously on day 1 every 2-week cycle for up to 2 years. Chemotherapy was administrated intravenously every 2-week by cisplatin (50 mg/m^2^ on day 1 for up to 8-cycle) and 5-fuorouracil (1,200 mg/m^2^ on days 1 and 2 of each cycle for up to 12-cycle). Patients would be treated with second-line treatments until disease progression or intolerable toxicities, which mainly included immunotherapy (camrelizumab or tislelizumab), chemotherapy (docetaxel) and best supportive care (**Supplementary Materials**). In ASTRUM-007 trial, a total of 95 (52%) patients in the chemotherapy group and 139 (38%) in the serplulimab plus chemotherapy group received subsequent anti-cancer treatments (10). The proportion of patients received subsequent therapies in each group was shown in **Table 1**.

**Table 1:** Model parameters and the range of the sensitivity analysis.

Parameters	Base Case	Range		Distribution	Source
		Minimum	Maximum		
Cost inputs (US $)					
Serplulimab (100 mg)	882.18	705.74	1058.62	Gamma	(12)
Cisplatin (10 mg)	1.47	1.18	1.77	Gamma	(12)
Fluorouracil (250 mg)	8.51	6.81	10.22	Gamma	(12)
Camrelizumab (200 mg)	462.25	369.80	554.69	Gamma	(12)
Tislelizumab (100mg)	228.91	183.13	274.69	Gamma	(12)
Docetaxel (20mg)	13.94	11.15	16.73	Gamma	(12)
BSC	182.23	145.78	218.68	Gamma	(13)
Routine follow-up cost	73.72	58.98	88.47	Gamma	(13)
Laboratory tests and radiological examinations	357.34	285.87	428.81	Gamma	(13)
Hospitalization expense	19.86	15.89	23.83	Gamma	(14)
Cost of AEs per unit					
Anemia	336.63	269.30	403.95	Gamma	(15)
Neutropenia	454.26	363.41	545.11	Gamma	(13)
Leukopenia	454.26	363.41	545.11	Gamma	(13)
Thrombocytopenia	1523.82	1219.06	1828.58	Gamma	(16)
Vomiting	101.15	80.92	121.38	Gamma	(15)
Hyponatraemia	3223.00	2578.40	3867.60	Gamma	(16)
Hypokalemia	3000.00	2400.00	3600.00	Gamma	Assumption
Utility inputs					
Utility of PFS	0.75	0.60	0.90	Beta	(17)
Utility of PD	0.60	0.48	0.72	Beta	(17)
AEs disutility					
Anemia	0.07	0.06	0.09	Beta	(18)
Neutropenia	0.20	0.16	0.24	Beta	(19)
Leukopenia	0.20	0.16	0.24	Beta	(19)
Thrombocytopenia	0.11	0.09	0.13	Beta	(20)
Vomiting	0.13	0.10	0.15	Beta	(19)
Hyponatraemia	0.04	0.03	0.05	Beta	(21)
Hypokalemia	0.04	0.03	0.05	Beta	Assumption
Risk of ≥ grade 3 AEs (%)					
Serplulimab plus chemotherapy					
Anemia	17.54	14.03	21.05	Beta	(10)
Leukopenia	11.26	9.01	13.51	Beta	(10)
Neutropenia	18.59	14.87	22.30	Beta	(10)
Thrombocytopenia	3.93	3.14	4.71	Beta	(10)
Vomiting	3.14	2.51	3.77	Beta	(10)
Hyponatremia	4.71	3.77	5.65	Beta	(10)
Hypokalemia	3.66	2.93	4.40	Beta	(10)
Chemotherapy					
Anemia	20.24	16.19	24.29	Beta	(10)
Leukopenia	6.55	5.24	7.86	Beta	(10)
Neutropenia	17.26	13.81	20.71	Beta	(10)
Hypokalemia	3.57	2.86	4.29	Beta	(10)
Proportion of Subsequent treatment (%)					
Chemotherapy					
Immunotherapy	33.33	26.67	40.00	Beta	(10)
Chemotherapy	20.00	16.00	24.00	Beta	(10)
BSC	46.67	37.33	56.00	Beta	(10)
Serplulimab plus chemotherapy					
Immunotherapy	17.39	13.91	20.87	Beta	(10)
Chemotherapy	20.00	16.00	24.00	Beta	(10)
BSC	62.61	50.09	75.13	Beta	(10)
Others					
Discount rate (%)	5.00	0.00	8.00	Beta	(22)
Patient weight (kg)	65.00	52.00	78.00	Gamma	(13)
Body surface area (m^2^)	1.72	1.38	2.06	Gamma	(13)

BSC, Best supportive care; AEs, adverse events; PFS, progression-free survival; PD, progressive disease.

### Model construction

**Figure 1:**
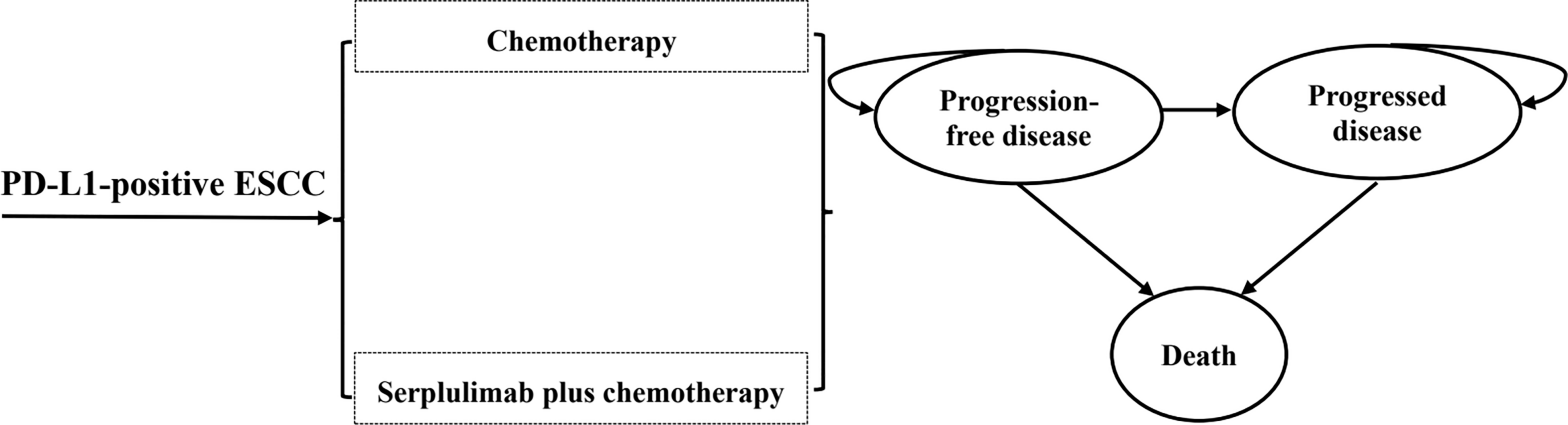
The structure of the partitioned survival model.

A partitioned survival model was constructed with three exclusive health states (PFS, progression-disease [PD], and death) to portray disease progression and treatment efficacy (**Figure 1**). The proportion of progression-free patients derived directly from the PFS curve, while the proportion of patients in the death state as 1 minus the OS curve. With regard to the PD state, its proportion was calculated as the difference between the PFS and OS curves (23).The time horizon of 10 years was adequate to ensure that ESCC patients completely entered the terminal state. The cycle length was 2-week to accommodate the treatment and follow-up regimens. Treatment strategies were compared in terms of overall costs, quality-adjusted life years (QALYs), and incremental cost-effectiveness ratios (ICERs; the incremental cost between two treatments per additional QALY gained). According to China Guidelines for Pharmacoeconomic Evaluations, half-cycle correlation and 5% annual discount rate were applied to costs and health outcomes (22). All costs were adjusted to 2023 prices with the local Consumer Price Index and converted into US dollars (1$=6.33 CNY). As recommended by the World Health Organization (24) and China Guidelines for Pharmacoeconomic Evaluations (22), 3 times per capita gross domestic product (GDP) of China in 2022 ($40,587.59) was implemented as the willingness-to-pay (WTP) threshold to judge the cost-effectiveness of serplulimab plus chemotherapy.

### Clinical inputs

Since individual patient data was unavailable, GetData Graph Digitizer 2.26 (http://www.getdata-graph-digitizer.com/) was applied to extract PFS and OS data points from the Kaplan-Meier curves reported in the ASTRUM-007 trial (**Supplementary Tables S2, S3**). To optimally extrapolate the lifetime survival outcome, Exponential, Weibull, Log-logistic, Log-normal, and Gompertz distributions were used to fit the individual-level data (25). The selection of optimal parametric distribution was based on clinical plausibility, Akaike Information Criterion, Bayesian Information Criterion and visual examination (26). The estimated shape parameters (γ) and scale parameters (λ) were summarized in **Supplementary Table S4**. Long-term survival data were presented in **Supplementary Table S5, Table S6** and **Figure S1**.

### Cost inputs

Only direct medical costs were considered, including drug costs, subsequent therapy, hospitalization expense, routine follow-up and examinations, and costs for the management of AEs. The prices of serplulimab, camrelizumab and tislelizumab were derived from lowest winning bids. Other drug costs were calculated from the average winning bids in 2023 of the YAOZHI database (https://data.yaozh.com/), which aggregated the latest price data around the country (12). Our prices were accessed on February 2, 2023. To determine the dosage and expenditure of therapeutic agents, the default height of 165 cm and body weight of 65kg, resulting in a body surface area (BSA) of 1.72 m^2^ were assumed for the Chinese ESCC patients (13). Other costs were retrieved from previously published literatures, such as best supportive care, routine follow-up, hospitalization, laboratory tests and radiological examinations (13, 14). In both groups, the frequency of laboratory work, computed tomography or magnetic resonance imaging examination were further determined according to Guidelines of Chinese Society of Clinical oncology (27). Grade 3 or above AEs with an incidence of greater than 3% were considered, including anemia, neutropenia, leukopenia, thrombocytopenia, vomiting, hyponatraemia and hypokalemia (13, 15, 16). All cost-related inputs were shown in **Table 1**.

### Health state utility

Each health state was assigned a utility anchored in 0 (death) and 1 (perfect health) in this partitioned survival model. Health state utilities for the PFS and PD health states were estimated from patient-level EQ-5D-3L data from the RAINBOW trial due to the absence of relevant data from the ASTRUM-007 trial (17). The utility values for PFS and PD states associated with advanced ESCC were 0.75 and 0.60, respectively, have been employed in multiple economic evaluations (13, 28, 29). Additionally, utility decrements caused by grade 3 or above treatment-related AEs were considered by multiplying the duration-adjusted disutilities by the incidence of AEs. The disutilities were also extracted from published studies (18–21). All utility-related parameters were shown in **Table 1**.

### Subgroup and scenario and analyses

In subgroup analyses, we analyzed the cost-effectiveness of serplulimab plus chemotherapy in the first-line treatment for advanced ESCC patients with PD-L1 expression level of 1 ≤ CPS < 10, and with PD-L1 CPS ≥ 10 through the methods of base-case analysis, respectively.

In scenario analyses, the shorter time horizons (2, 5 and 8 years) were used to investigate the impact on the model results. Furthermore, we explored the influence of various price-reduction levels for serplulimab on ICERs. At the same time, we assessed the probability of serplulimab plus chemotherapy being cost-effective by assuming a 70% price reduction of serplulimab, which was comparable to the prices of camrelizumab, sintilimab, tislelizumab and toripalimab.

### Sensitivity analyses

One-way and probabilistic sensitivity analyses (PSA) were conducted for all key variables to estimate the robustness of our results. In the one-way sensitivity analyses, the plausible range of each parameter was either based on the reported 95%CI or calculated by assuming a 20% deviation from the base-case value. The range of discount rate was set as 0%-8% in line with China Guidelines for Pharmacoeconomic Evaluations (22). The results were presented in the form of tornado diagrams. For the PSA, 10,000 Monte Carlo simulations were generated by simultaneously sampling all parameters from the pre-specified statistical distributions. Gamma distributions were selected for cost inputs, and beta distributions were used for utility values and probabilities (30). The scatter plot and cost-effectiveness acceptability curves (CEAC) based on the results from 10,000 iterations were plotted to illustrate the probability of serplulimab plus chemotherapy would be considered cost-effective at various WTP thresholds.

## Results

### Base-case results

The base-case results were shown in **Table 2**. Compared with chemotherapy, patients received serplulimab plus chemotherapy yielded an additional 0.27 QALY at an incremental cost of $33,460.86. The ICER was $124,483.07 per QALY gained, which was significantly higher than the WTP threshold. When only focused on PFS period, serplulimab plus chemotherapy was associated with 0.19 greater QALYs compared to chemotherapy at an additional cost of $34,204.22, which had an ICER of $176,431.72 per QALY gained.

**Table 2:** Results of base-case analysis and subgroup analysis.

Parameters	All patients		Patients with 1 ≤ CPS < 10		Patients with CPS ≥ 10	
	C	S+C	C	S+C	C	S+C
OS						
Total cost ($)	4,999.71	38,460.57	4,578.46	34,941.82	5,415.47	50,871.24
QALYs	0.83	1.10	0.76	0.98	0.87	1.30
ICER ($/QALY)		124,483.07		134,637.42		105,589.71
Only PFS						
Total cost ($)	2,787.42	36,991.64	2,620.99	33,653.44	3,504.03	49,406.45
QALYs	0.38	0.57	0.36	0.52	0.48	0.77
ICER ($/QALY)		176,431.72		190,015.77		155,496.70

S+C, Serplulimab plus chemotherapy; C, chemotherapy; QALYs, quality-adjusted life years, ICER, incremental cost-effectiveness ratios.

### Subgroup and scenario analyses results

In subgroup analyses, the ICERs of serplulimab plus chemotherapy versus chemotherapy were $134,637.42 and $105,589.71 per QALY gained in patients with PD-L1 expression level of 1 ≤ CPS < 10 and CPS ≥ 10, respectively (**Table 2**). When only focused on PFS period, serplulimab plus chemotherapy compared with chemotherapy resulted in the ICERs of $190,015.77 and $155,496.70 per QALY gained in patients with PD-L1 1 ≤ CPS < 10 and CPS ≥ 10, respectively.

**Figure 2:**
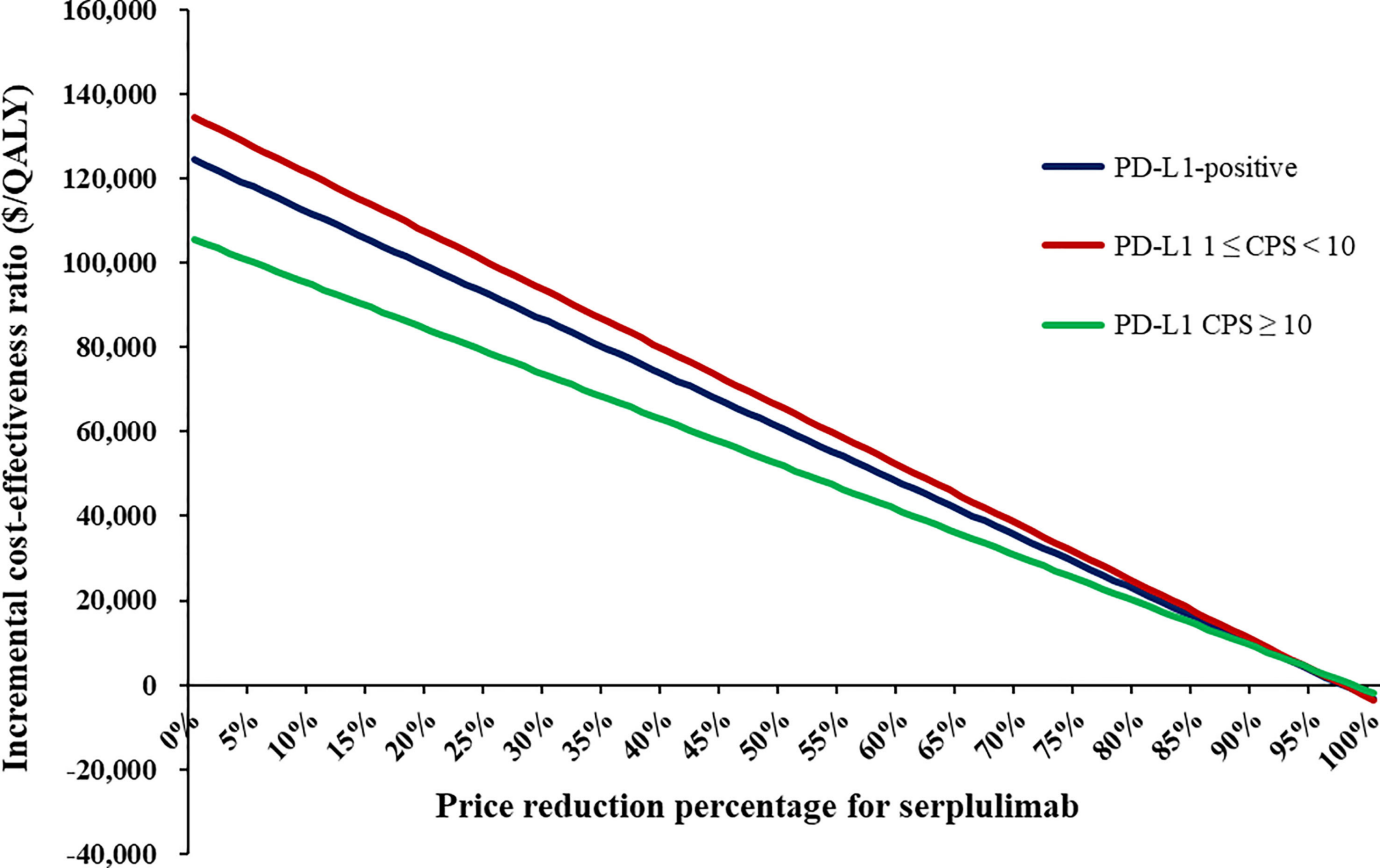
The impact of the price reduction of serplulimab on ICERs.

In scenario analyses, as the time horizon lengthened, the ICERs of serplulimab plus chemotherapy gradually decreased, but were consistently higher than the WTP threshold (**Supplementary Table S7**). When the price of serplulimab was decreased by 66%, 69% and 61%, respectively, the ICERs of serplulimab plus chemotherapy versus chemotherapy were lower than the WTP thresholds for advanced ESCC patients with PD-L1-positive, PD-L1 1 ≤ CPS < 10 and CPS ≥ 10 (**Figure 2**).

### Sensitivity analyses results

**Figure 3:**
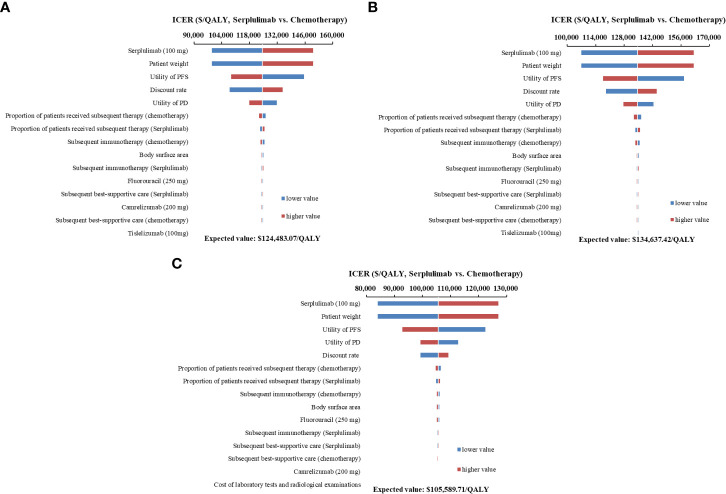
Tornado diagrams of one-way sensitivity analyses. **(A)** all PD-L1-positive advanced esophageal squamous cell carcinoma patients; **(B)** Patients with PD-L1 1 ≤ CPS < 10; **(C)** Patients with PD-L1 CPS ≥ 10) ICER, incremental cost-effectiveness ratios; PFS, progression-free survival; PD, progressive disease.

**Figure 4:**
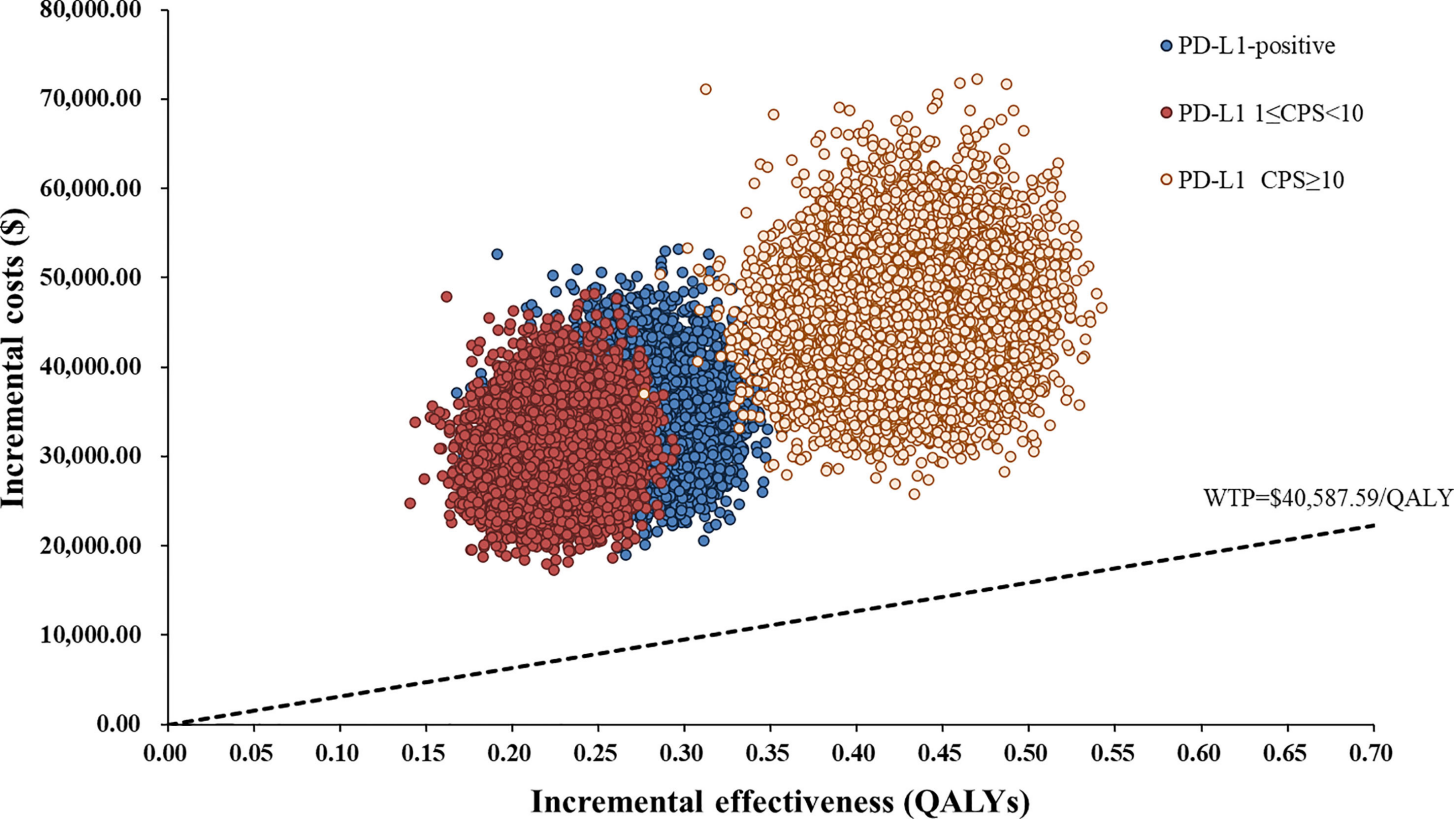
Scatter Plot of the ICER between serplulimab plus chemotherapy and chemotherapy. WTP, willingness-to-pay; QALY, quality-adjusted life years.

**Figure 5:**
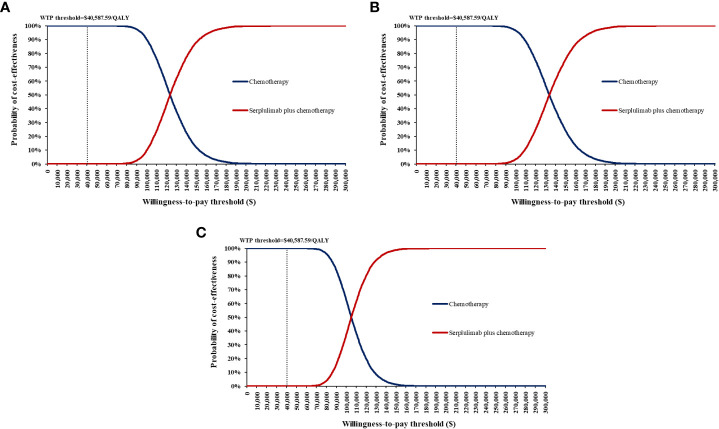
Cost-effectiveness acceptability curves for serplulimab plus chemotherapy versus chemotherapy. **(A)** all PD-L1-positive advanced esophageal squamous cell carcinoma patients; **(B)** Patients with PD-L1 1 ≤ CPS < 10; **(C)** Patients with PD-L1 CPS ≥ 10) WTP, willingness-to-pay.

One-way sensitivity analyses demonstrated that the price of serplulimab, patient weight, utility of PFS and PD, and discount rate had the most significant impact on the base-case results (**Figure 3**). The ICERs were consistently higher than the WTP threshold with the alterations in all uncertainty parameters. Results of PSA were shown in **Figure 4**. According to the scatter plot, compared with chemotherapy, all scatter points were located in the North-East quadrant and above the WTP threshold. At the WTP threshold of 3 times per capita GDP in China, the CEAC revealed that a 0% probability of serplulimab plus chemotherapy being cost-effective in various groups (PD-L1-positive, PD-L1 1 ≤ CPS < 10 and CPS ≥ 10 patients) (**Figure 5**). When serplulimab was reduced to 30% of the current price ($264.65 per 100mg), the probabilities of serplulimab plus chemotherapy being cost-effective were 81.42%, 67.74% and 96.75% in advanced ESCC patients with PD-L1-positive, PD-L1 1≤CPS<10 and CPS≥10, respectively (**Supplementary Figures S2, S3**).

## Discussion

To our knowledge, this is the first study to appraise the cost-effectiveness of serplulimab plus chemotherapy in the first-line treatment of patients with PD-L1-positive advanced ESCC from the Chinese healthcare system perspective. Compared with chemotherapy, serplulimab plus chemotherapy achieved additional 0.27, 0.23 and 0.43 QALYs with marginal $33,460.86, $30,363.35 and $45,455.77, which resulted in the ICERs of $124,483.07, $134,637.42 and $105,589.71 per QALY gained in advanced ESCC patients with PD-L1-positive, PD-L1 1 ≤ CPS < 10 and CPS ≥ 10, respectively. At the current prices and WTP threshold, serplulimab plus chemotherapy might not be cost-effective compared to chemotherapy. The base-case results were upheld by the subgroup, scenario and sensitivity analyses.

The price of serplulimab, patient weight, utility values and discount rate were the most influential parameters, but alterations in each parameter did not alter the conclusion. The price of serplulimab was extremely expensive compared to other domestic PD-1 inhibitors for the treatment of advanced ESCC patients, which became an essential parameter in dominating cost-effectiveness. Therefore, substantial price reductions or generous patient assistance programs would contribute to increased affordability of patients. Of note, when the price of serplulimab was reduced by 70%, the probability of serplulimab plus chemotherapy being cost-effective increased from 0% to 81.42%, 67.74% and 96.75%, respectively, in advanced ESCC patients with PD-L1-positive, PD-L1 1 ≤ CPS < 10 and CPS ≥ 10. When the price of serplulimab was decreased by 98%, 98% and 99%, respectively, serplulimab plus chemotherapy would be absolutely dominant regimens over chemotherapy (ICER < 0) for advanced ESCC patients with PD-L1-positive, PD-L1 1 ≤ CPS < 10 and CPS ≥ 10. The primary reason was that the higher proportion of patients in the chemotherapy group received second-line immunotherapy compared to the serplulimab plus chemotherapy group. Our results were consistent with previously published studies (13, 31) that patient weight was a significant parameter because serplulimab was administered based on body weight, revealing that serplulimab plus chemotherapy would be unfavorable in overweight or obese patients because of more dosages and expenditures required.

Serplulimab received its first approval on 25 March 2022 in China, and the economic evidence was relatively limited (9). Zhu et al. (32) estimated the cost-effectiveness of serplulimab plus chemotherapy as the first-line treatment for extensive-stage small-cell lung cancer (ES-SCLC) from a payer perspective in China based on the ASTRUM-005 trial (7). The results demonstrated that the probability of serplulimab plus chemotherapy being cost-effective was 91.6% compared with mono-chemotherapy at the WTP threshold of 3 times per capita GDP of China in 2021 (32). Another study by Shao et al. (33) showed that serplulimab might be a valuable and cost-effective regime as first-line therapy for ES-SCLC patients in both the United States and China. However, the advantage of cost-effectiveness has not been identified in PD-L1-positive advanced ESCC patients, which primarily attributed to better survival improvements of serplulimab plus chemotherapy in extensive-stage small-cell lung cancers.

PD-L1 expression was enriched in ESCC patients, ranging from 15%-83% in tumor cells and 13%-31% in immune cells, which greatly increased tumor susceptibility in patients receiving immune checkpoint inhibitors (34, 35). Prior economic evidences indicated that PD-1 inhibitors were potentially sensitive to PD-L1-positive ESCC patients, with higher survival benefits and health outcomes compared with the overall population (13, 16, 31, 36, 37). Whether overall or PD-L1-positive advanced ESCC patients, the probability of nivolumab or pembrolizumab plus chemotherapy being cost-effective were 0% compared with chemotherapy (13, 31, 36). Shao et al. demonstrated that the probability of sintilimab plus chemotherapy being cost-effective in PD-L1-positive advanced ESCC patients would be increased by 30% compared to PD-L1-negative patients (16). In this study, we found that higher PD-L1 expression levels were associated with better cost-effectiveness, indicating that patients with advanced ESCC should receive appropriate treatment regimens in accordance with PD-L1 expression levels in clinical management.

Currently, numerous studies are targeted on the economic evaluations of immunotherapies for advanced ESCC patients and warrant discussion. At the current price and WTP threshold, domestic PD-1 inhibitors, such as camrelizumab, sintilimab, tislelizumab and toripalimab, were cost-effective options as first- or second-line treatment for patients with advanced ESCC in China (18, 29, 38–40). The dynamic adjustment mechanism of the national medical insurance catalog has played a predominant role in this situation. Numerous anti-cancer innovative drugs have been substantially reduced in price by approximately 70%, which has greatly improved the accessibility and affordability for patients (41, 42). Intensive concern regarding the affordability of treatment regimens is currently shared by both patients and clinicians (32). Taking cost-effectiveness into considerations in clinical practice and healthcare decisions is crucial for clinicians and policy-makers to address the financial burdens of patients and allocate limited healthcare resources. Serplulimab has not yet successfully undergone national health insurance negotiations, but its significant clinical benefits have emerged as a potential treatment option for health systems and patients (10).

There were several limitations in this study. First, since the sample size of the ASTRUM-007 trial was relatively small and actual survival data were not available, we employed reconstructed and extrapolated individual patient data to construct the partitioned survival model. Further studies based on long-term efficacy data or large samples of real-world evidence would be needed to validate the results. Second, The utilities and disutilities in this analysis primarily derived from the published literature, because of the absence of quality-of-life data in the ASTRUM-007 trial. According to one-way sensitivity analyses, the health state utilities did not influence on the conclusions. Third, subsequent strategies after the progression in the first-line treatment were based on the ASTRUM-007 trial, which might be inconsistent with the actual clinical practice situation. Forth, the costs and disutilities of grade 1-2 treatment-related serious AEs were excluded from this study, which might overestimate the cost-effectiveness of serplulimab plus chemotherapy, although one-way sensitivity analyses performed that only minimal impact on the model results. Fifth, other immunotherapies, such as camrelizumab, nivolumab, pembrolizumab, sintilimab and toripalimab, which had significant survival benefits for overall and PD-L1-positive advanced ESCC patients, were not included in this economic evaluation.

## Conclusion

In summary, from the Chinese healthcare system perspective, serplulimab plus chemotherapy might not be considered cost-effective in the first-line treatment for PD-L1-positive advanced ESCC patients despite the extension of PFS and OS. Substantial price reductions could improve cost-effectiveness of serplulimab. When the prices of serplulimab were decreased by 66%, 69% and 61%, respectively, serplulimab plus chemotherapy would be cost-effective in advanced ESCC patients with PD-L1-positive, PD-L1 1 ≤ CPS < 10 and CPS ≥ 10.

## Data availability statement

The original contributions presented in the study are included in the article/**Supplementary Material**. Further inquiries can be directed to the corresponding author.

## Author contributions

SXL, NJ, and LD were responsible for study design, model building and statistical analysis. SXL and NJ prepared the manuscript. SPL and LD searched literatures and collected data. All authors critically reviewed the model structure, verified results and revised the manuscript. All authors contributed to the article and approved the submitted version.
