# Supplementary material for: Cost-effectiveness analysis of serplulimab plus chemotherapy in the first-line treatment for PD-L1-positive esophageal squamous cell carcinoma in China
Source: Front Immunol. 2023 May 4;14:1172242. doi: 10.3389/fimmu.2023.1172242 (PMC10192749; doi:10.3389/fimmu.2023.1172242)
Supplement: Supplementary file 1 [file DataSheet_1.pdf]

Cost-effectiveness analysis of serplulimab plus chemotherapy in the first-line treatment for PD-L1-positive esophageal squamous cell carcinoma in China

Shixian Liu[1,2,3†], Nana Jiang[4†], Lei Dou[1,2,3*], and Shunping Li[1,2,3]

[1]Centre for Health Management and Policy Research, School of Public Health, Cheeloo College of Medicine, Shandong University, Jinan, China,

[2]NHC Key Laboratory of Health Economics and Policy Research (Shandong University), Jinan, China,

[3]Center for Health Preference Research, Shandong University, Jinan, China,

[4]Department of Maternal and Child Health, School of Public Health, Cheeloo College of Medicine, Shandong University, Jinan, China.

[*]Correspondence:

Lei Dou
doulei@sdu.edu.cn

[†]These authors have contributed equally to this work.

Treatment Strategies

PFS state:

1. **Chemotherapy:** Cisplatin (50 mg/m^2 on day 1 every 2 weeks for up to 8-cycle) and 5-fuorouracil (1,200 mg/m^2 on days 1-2 every 2 weeks for up to 12-cycle).
2. **Serplulimab plus chemotherapy:** Serplulimab (3 mg/kg on day 1 every 2 weeks), plus Cisplatin (50 mg/m^2 on day 1 every 2 weeks for up to 8 cycles) and fluorouracil (1,200 mg/m^2 on days 1-2 every 2 weeks for up to 12 cycles).

PD state:

1. **Camrelizumab:** Camrelizumab (200 mg intravenously on day 1 every 2 weeks).
2. **Tislelizumab:** Tislelizumab (200 mg intravenously on day 1 every 3 weeks).
3. **Docetaxel:** Docetaxel (75 mg/m^2 intravenously on day 1 every 3 weeks).
4. **Best supportive care**.

Ref:

1. Song Y, Zhang B, Xin D, Kou X, Tan Z, Zhang S, et al. First-Line Serplulimab or Placebo Plus Chemotherapy in Pd-L1-Positive Esophageal Squamous Cell Carcinoma: A Randomized, Double-Blind Phase 3 Trial. Nat Med (2023) 29(2):473-482. doi: 10.1038/s41591-022-02179-2.
2. Huang J, Xu J, Chen Y, Zhuang W, Zhang Y, et al. Camrelizumab versus investigator's choice of chemotherapy as second-line therapy for advanced or metastatic oesophageal squamous cell carcinoma (ESCORT): a multicentre, randomised, open-label, phase 3 study. Lancet Oncol (2020) 21(6):832-842. doi: 10.1016/S1470-2045(20)30110-8.
3. Shen L, Kato K, Kim SB, Ajani JA, Zhao K, et al. Tislelizumab Versus Chemotherapy as Second-Line Treatment for Advanced or Metastatic Esophageal Squamous Cell Carcinoma (RATIONALE-302): A Randomized Phase III Study. J Clin Oncol (2022) 40(26):3065-3076. doi: 10.1200/JCO.21.01926.
4. Ford HE, Marshall A, Bridgewater JA, Janowitz T, Coxon FY, et al. Docetaxel versus active symptom control for refractory oesophagogastric adenocarcinoma (COUGAR-02): an open-label, phase 3 randomised controlled trial. Lancet Oncol (2014) 15(1):78-86. doi: 10.1016/S1470-2045(13)70549-7.
5. Liu S, Dou L, Wang K, Shi Z, Wang R, Zhu X, et al. Cost-Effectiveness Analysis of Nivolumab Combination Therapy in the First-Line Treatment for Advanced Esophageal Squamous-Cell Carcinoma. Front Oncol (2022) 12:899966. doi: 10.3389/fonc.2022.899966.

Supplementary Materials

Supplementary Table S1 | CHEERS Checklist 2022.

Supplementary Table S2 | Individual patient data for chemotherapy (PFS).

Supplementary Table S3 | Individual patient data for chemotherapy (OS).

Supplementary Table S4 | Summary of statistical goodness-of-fit of Kaplan-Meier curves.

Supplementary Table S5 | Long-term PFS data.

Supplementary Table S6 | Long-term OS data.

Supplementary Figure S1 | The reconstructed Kaplan-Meier PFS and OS curves.

Supplementary Table S7 | Scenario analysis results of various time horizons.

Supplementary Figure S2 | Scatter Plot of the ICER between serplulimab plus chemotherapy and chemotherapy (serplulimab at 30% of current price).

Supplementary Figure S3 | Cost-effectiveness acceptability curves for serplulimab plus chemotherapy versus chemotherapy (serplulimab at 30% of current price).

Supplementary Table S1 | CHEERS Checklist 2022.

**Section**	**Item No**	**Guidance for reporting**	**Reported**
**Title**			
Title	1	Identify the study as an economic evaluation and specify the interventions being compared	Yes
**Abstract**			
Abstract	2	Provide a structured summary that highlights context, key methods, results, and alternative analyses	Yes
**Introduction**			
Background and objectives	3	Give the context for the study, the study question, and its practical relevance for decision making in policy or practice	Yes
**Methods**			
Health economic analysis plan	4	Indicate whether a health economic analysis plan was developed and where available	Yes
Study population	5	Describe characteristics of the study population (such as age range, demographics, socioeconomic, or clinical characteristics)	Yes
Setting and location	6	Provide relevant contextual information that may influence findings	Yes
Comparators	7	Describe the interventions or strategies being compared and why chosen	Yes
Perspective	8	State the perspective(s) adopted by the study and why chosen	Yes
Time horizon	9	State the time horizon for the study and why appropriate	Yes
Discount rate	10	Report the discount rate(s) and reason chosen	Yes
Selection of outcomes	11	Describe what outcomes were used as the measure(s) of benefit(s) and harm(s)	Yes
Measurement of outcomes	12	Describe how outcomes used to capture benefit(s) and harm(s) were measured	Yes
Valuation of outcomes	13	Describe the population and methods used to measure and value outcomes	Yes
Measurement and valuation of resources and costs	14	Describe how costs were valued	Yes
Currency, price date, and conversion	15	Report the dates of the estimated resource quantities and unit costs, plus the currency and year of conversion	Yes
Rationale and description of model	16	If modelling is used, describe in detail and why used. Report if the model is publicly available and where it can be accessed	Yes
Analytics and assumptions	17	Describe any methods for analysing or statistically transforming data, any extrapolation methods, and approaches for validating	Yes

		any model used	
Characterising heterogeneity	18	Describe any methods used for estimating how the results of the study vary for subgroups	Yes
Characterising distributional effects	19	Describe how impacts are distributed across different individuals or adjustments made to reflect priority populations	Yes
Characterising uncertainty	20	Describe methods to characterise any sources of uncertainty in the analysis	Yes
Approach to engagement with patients and others affected by the study	21	Describe any approaches to engage patients or service recipients, the general public, communities, or stakeholders (such as clinicians or payers) in the design of the study	Yes
**Results**			
Study parameters	22	Report all analytic inputs (such as values, ranges, references) including uncertainty or distributional assumptions	Yes
Summary of main results	23	Report the mean values for the main categories of costs and outcomes of interest and summarise them in the most appropriate overall measure	Yes
Effect of uncertainty	24	Describe how uncertainty about analytic judgments, inputs, or projections affect findings. Report the effect of choice of discount rate and time horizon, if applicable	Yes
Effect of engagement with patients and others affected by the study	25	Report on any difference patient/service recipient, general public, community, or stakeholder involvement made to the approach or findings of the study	Yes
**Discussion**			
Study findings, limitations, generalisability, and current knowledge	26	Report key findings, limitations, ethical or equity considerations not captured, and how these could affect patients, policy, or practice	Yes
Other relevant information Source of funding	27	Describe how the study was funded and any role of the funder in the identification, design, conduct, and reporting of the analysis	Yes
Conflicts of interest	28	Report authors conflicts of interest according to journal or International Committee of Medical Journal Editors requirements	Yes

Supplementary Table S2 | Individual patient data for chemotherapy (PFS).

No	PD-L1-positive				PD-L1 1 ≤ CPS < 10				PD-L1 CPS ≥ 10			
	Chemotherapy		Serplulimab		Chemotherapy		Serplulimab		Chemotherapy		Serplulimab	
	Time	rate	Time	rate	Time	rate	Time	rate	Time	rate	Time	rate
1	0.00	100.00	0.00	100.00	0.00	100.00	0.00	100.00	0.00	100.00	0.00	100.00
2	0.25	99.49	0.28	99.49	0.81	99.23	0.31	99.49	0.28	98.71	0.79	99.49
3	0.48	99.23	0.48	99.23	1.07	98.46	0.53	98.72	0.66	97.43	1.17	98.97
4	0.71	98.72	0.71	98.97	1.20	97.69	0.92	97.95	1.04	95.63	1.25	98.20
5	0.79	98.21	0.97	98.46	1.30	93.08	0.99	97.69	1.25	94.34	1.30	97.43
6	1.04	97.44	1.04	97.18	1.48	91.54	1.04	96.15	1.35	90.49	1.35	91.26
7	1.22	96.41	1.20	96.15	1.55	90.26	1.17	95.38	1.50	87.92	1.40	90.49
8	1.27	94.62	1.27	94.62	1.63	89.23	1.20	93.33	1.65	86.38	1.50	88.95
9	1.35	92.82	1.32	93.59	1.86	87.95	1.25	92.31	1.81	84.83	1.68	88.69
10	1.37	91.28	1.37	91.28	1.96	87.18	1.30	91.28	2.09	83.55	1.70	88.43
11	1.55	88.72	1.48	89.23	2.01	85.64	1.40	90.26	2.11	82.26	1.81	87.66
12	1.75	87.44	1.58	88.21	2.57	85.13	1.50	88.72	2.26	80.72	1.96	87.15
13	1.88	86.41	1.75	87.18	2.62	84.10	1.60	87.18	2.31	79.18	2.62	86.38
14	1.98	85.90	1.86	86.41	2.70	82.56	1.65	86.15	2.75	73.78	2.67	86.12
15	2.06	85.38	2.01	86.15	2.72	80.77	1.83	85.64	2.87	72.24	2.75	85.09
16	2.14	84.36	2.21	85.90	2.80	78.72	2.29	85.13	2.95	70.44	2.77	84.32
17	2.29	83.85	2.49	85.64	2.90	76.92	2.57	84.62	3.03	69.41	2.92	83.55
18	2.34	83.08	2.54	85.13	2.95	75.90	2.67	83.59	3.20	67.87	3.00	82.78
19	2.57	82.31	2.64	84.62	3.00	74.10	2.75	78.97	3.69	66.58	3.10	82.01
20	2.64	81.03	2.67	84.10	3.28	71.79	2.87	76.92	3.87	64.52	3.76	81.49
21	2.72	80.51	2.72	83.33	3.84	69.49	3.18	75.90	4.02	62.98	3.81	80.72
22	2.75	79.23	2.75	82.31	3.94	67.44	3.28	75.38	4.12	58.61	3.92	79.43
23	2.82	76.41	2.82	81.54	4.02	64.87	3.48	74.36	4.30	55.53	4.04	77.12
24	2.87	75.13	2.92	80.51	4.04	63.85	3.81	73.85	4.32	53.73	4.12	73.52
25	2.98	72.82	3.03	79.23	4.07	62.56	3.99	73.33	4.88	52.19	4.15	72.49
26	3.08	71.79	3.23	78.72	4.15	60.26	4.04	72.31	5.09	50.64	4.35	71.98
27	3.23	70.26	3.48	77.95	4.27	58.97	4.07	71.03	5.21	48.59	4.70	71.21
28	3.71	69.49	3.79	77.18	4.32	54.87	4.09	67.69	5.42	45.24	4.78	70.95
29	3.81	67.95	3.94	76.41	4.45	52.31	4.15	65.90	5.49	43.70	4.81	70.44
30	3.92	65.90	3.97	75.90	4.58	50.77	4.25	65.13	5.52	41.90	5.26	68.38
31	4.02	65.13	4.02	74.36	5.32	48.21	4.30	62.56	5.72	39.59	5.29	66.32
32	4.09	62.56	4.04	73.33	5.42	46.67	4.37	62.05	5.98	38.05	5.49	62.21
33	4.12	60.51	4.09	72.05	5.47	45.38	4.50	61.03	6.38	35.99	5.57	61.44
34	4.17	57.95	4.15	71.03	5.52	41.03	4.81	60.51	6.76	34.19	5.67	60.15
35	4.35	54.62	4.17	69.49	5.54	38.46	5.14	59.74	6.82	32.39	5.72	57.07
36	4.45	53.33	4.30	68.21	5.62	34.36	5.34	58.97	6.87	30.59	6.05	56.30
37	4.58	52.31	4.32	67.18	5.65	32.56	5.44	57.44	6.92	28.28	6.69	54.24
38	4.91	51.54	4.35	66.41	5.75	31.28	5.52	56.15	7.07	26.74	6.76	52.96

39	5.14	50.77	4.40	66.15	6.00	30.00	5.57	54.87	7.50	24.94	6.89	50.13
40	5.26	50.00	4.78	65.90	6.28	28.21	5.59	53.33	8.27	21.34	6.97	49.10
41	5.34	48.21	4.83	65.38	6.43	27.18	5.67	50.26	8.29	15.68	7.02	48.33
42	5.47	45.38	5.11	64.87	6.74	25.38	5.77	45.90	8.32	13.11	7.12	47.56
43	5.52	43.33	5.32	63.08	6.84	24.87	5.80	44.10	9.18	11.57	7.25	46.53
44	5.54	40.26	5.44	61.54	6.92	21.79	6.00	43.33	9.69	9.25	7.86	45.76
45	5.62	37.69	5.52	58.21	6.94	20.51	6.28	42.56	13.58	7.20	8.39	44.47
46	5.67	36.67	5.57	56.92	6.99	18.46	6.31	42.05	13.94	4.63	8.62	43.44
47	5.77	35.38	5.67	55.90	7.07	16.67	6.49	41.28			8.82	42.42
48	6.00	33.59	5.70	54.62	7.17	15.38	6.59	40.51			8.95	41.39
49	6.26	32.82	5.72	52.82	7.99	13.08	6.71	39.49			9.03	40.36
50	6.43	31.28	5.75	50.77	8.16	11.28	6.89	38.46			9.38	39.33
51	6.74	30.51	5.77	49.74	11.34	9.49	6.99	36.15			9.49	37.28
52	6.82	28.21	6.08	49.23			7.07	34.10			9.77	36.25
53	6.87	26.15	6.31	48.72			7.12	33.08			9.79	35.22
54	6.94	24.36	6.49	48.21			7.20	32.05			10.05	33.93
55	6.97	23.08	6.59	47.69			7.35	31.28			10.99	32.90
56	7.07	22.56	6.71	46.41			7.45	30.51			11.09	32.13
57	7.12	21.28	6.82	45.13			7.50	29.74			11.42	30.59
58	7.17	20.51	6.84	44.62			8.09	28.72			13.53	29.05
59	7.55	19.74	6.92	43.59			8.32	28.21			13.99	27.51
60	7.99	18.72	6.99	42.82			8.39	26.92			16.68	24.94
61	8.14	17.95	7.07	41.03			8.49	24.36			19.23	20.82
62	8.29	15.90	7.15	39.49			9.18	23.33			19.33	16.71
63	8.32	13.33	7.30	38.97			9.94	22.31				
64	8.34	12.31	7.35	38.46			11.09	21.28				
65	9.21	11.28	7.43	38.21			12.92	20.77				
66	9.74	10.26	7.48	37.69			13.05	20.00				
67	11.32	9.23	7.91	37.18			13.71	17.95				
68	13.63	8.21	8.09	36.67			13.81	16.92				
69	14.01	7.18	8.32	36.15			13.89	13.85				
70			8.39	35.64			20.29	10.77				
71			8.44	35.38								
72			8.47	34.10								
73			8.62	33.59								
74			8.88	32.82								
75			9.03	32.56								
76			9.10	32.05								
77			9.44	30.51								
78			9.51	30.26								
79			9.84	28.97								
80			10.10	27.95								
81			11.01	27.44								

82			11.09	27.18									
83			11.16	26.15									
84			11.47	25.64									
85			13.07	25.13									
86			13.58	24.36									
87			13.68	24.10									
88			13.81	22.56									
89			13.86	21.28									
90			13.99	20.51									
91			16.78	19.49									
92			19.28	17.44									
93			19.38	15.64									
94			20.27	13.33									

Supplementary Table S3 | Individual patient data for chemotherapy (OS).

No	PD-L1-positive				PD-L1 1 ≤ CPS < 10				PD-L1 CPS ≥ 10			
	Chemotherapy		Serplulimab		Chemotherapy		Serplulimab		Chemotherapy		Serplulimab	
	Time	rate	Time	rate	Time	rate	Time	rate	Time	rate	Time	rate
1	0.00	100.00	0.00	100.00	0.00	100.00	0.00	100.00	0.00	100.00	0.00	100.00
2	0.40	99.22	0.40	99.22	0.81	98.96	0.31	99.22	0.28	98.96	1.49	99.74
3	0.87	98.70	0.87	98.70	1.46	98.19	0.50	98.19	1.65	97.67	1.74	99.22
4	1.37	98.19	1.40	98.45	1.96	96.89	0.87	97.67	1.81	96.37	1.90	97.93
5	1.68	97.67	1.56	97.67	2.02	96.11	1.25	96.89	2.09	95.34	3.05	96.89
6	1.87	97.15	1.84	96.89	2.34	95.34	1.53	96.37	2.30	94.04	3.39	96.63
7	1.96	96.37	2.71	96.11	2.71	94.04	1.99	95.85	3.05	92.49	3.67	95.34
8	2.12	95.60	2.80	95.60	3.27	92.23	2.37	95.60	3.11	91.45	3.99	93.26
9	2.34	94.82	2.96	95.08	3.80	91.19	2.71	95.34	3.64	90.16	4.05	92.75
10	2.77	94.04	3.14	94.56	3.83	89.90	2.80	94.04	3.71	88.86	4.14	91.97
11	3.08	93.78	3.36	94.30	3.92	88.86	2.96	93.26	3.92	87.82	4.86	90.93
12	3.27	91.97	3.46	93.52	4.39	87.82	3.05	92.75	4.42	86.27	5.26	89.90
13	3.71	91.19	3.71	93.01	4.48	86.53	3.27	91.97	5.08	85.23	5.73	88.08
14	3.86	89.64	3.86	92.23	4.51	85.75	3.30	91.71	5.17	84.72	5.85	87.31
15	3.92	88.60	4.14	91.19	4.64	85.23	3.49	91.45	5.26	83.42	6.26	86.53
16	4.45	87.82	4.20	90.41	4.73	84.20	3.67	90.93	5.42	81.87	6.41	86.01
17	4.86	84.46	4.76	89.64	4.86	82.90	3.83	90.16	5.45	80.31	6.76	84.97
18	5.17	83.42	4.83	88.86	5.20	82.38	4.08	89.64	5.48	79.02	6.94	84.72
19	5.42	82.12	4.95	88.08	5.76	80.83	4.20	88.60	5.67	78.24	7.01	82.90
20	5.48	81.35	5.08	87.05	6.01	79.27	4.33	88.08	5.70	76.42	7.35	82.12
21	5.70	80.05	5.36	86.01	6.26	77.98	4.73	87.56	5.76	75.13	7.63	81.35
22	5.76	79.02	5.76	85.23	6.35	77.46	4.83	86.27	5.88	74.61	7.66	80.83
23	5.98	77.20	5.88	84.46	6.66	76.94	4.92	85.49	6.01	73.83	7.78	80.31
24	6.26	76.42	6.07	83.68	6.79	75.65	5.08	84.72	6.26	70.98	8.22	80.05
25	6.32	75.13	6.16	82.64	7.10	74.87	5.20	84.20	6.41	69.43	8.34	79.53
26	6.57	74.09	6.29	81.87	7.35	73.58	5.32	83.16	7.04	68.13	8.50	78.76
27	6.69	73.06	6.45	81.35	7.75	72.02	5.60	82.64	7.44	66.58	8.69	77.98
28	7.10	72.02	6.54	80.57	8.00	71.24	5.73	81.61	7.53	65.03	9.00	77.46
29	7.50	70.73	6.85	79.53	8.10	69.43	5.88	80.83	8.13	63.73	9.09	76.94
30	7.72	69.43	7.13	79.02	8.25	68.91	6.07	79.53	8.25	62.18	9.71	76.17
31	7.94	68.65	7.41	78.24	8.31	67.62	6.26	77.98	8.66	60.88	9.81	74.87
32	8.16	67.36	7.60	77.20	8.47	66.32	6.48	76.42	8.94	59.07	9.90	73.83
33	8.25	66.32	7.91	76.68	8.59	65.03	6.76	75.91	9.12	57.77	10.06	73.32
34	8.34	64.77	8.13	76.42	8.72	63.73	7.19	75.39	9.22	55.96	10.80	72.54
35	8.50	64.51	8.25	75.65	8.90	62.95	7.47	74.35	10.40	54.40	10.99	71.50
36	8.69	63.47	8.69	74.61	8.97	61.14	7.63	73.83	11.77	52.59	11.30	70.73
37	8.75	62.18	8.84	73.58	9.15	59.84	8.10	73.06	12.17	50.78	11.80	69.95
38	8.97	60.36	9.03	71.76	9.68	58.81	8.53	71.76	13.86	46.11	11.86	69.43

39	9.15	59.33	9.15	71.24	9.75	57.51	8.81	70.73	15.10	43.78	11.92	68.91
40	9.31	58.29	9.65	69.95	10.15	54.66	8.87	69.69	15.88	41.19	12.55	67.88
41	9.71	56.99	9.78	69.43	10.34	53.37	8.94	68.13	17.69	38.08	12.61	67.10
42	10.18	55.96	9.84	68.39	10.55	51.55	9.06	67.36	18.15	34.72	12.67	65.80
43	10.31	54.66	10.06	67.10	10.59	50.26	9.50	66.84	22.45	26.17	12.73	65.03
44	10.40	53.89	10.46	66.32	11.40	48.70	9.68	66.32	25.16	12.95	12.77	64.51
45	10.52	53.11	10.55	66.06	11.80	46.89	9.75	65.03			12.92	63.99
46	10.62	52.33	10.68	65.03	12.55	45.34	9.93	62.95			13.08	62.95
47	11.40	51.55	10.96	63.99	13.08	43.78	10.03	62.44			13.67	61.92
48	11.80	49.48	11.30	63.47	13.76	41.97	10.31	61.66			14.29	60.88
49	12.17	48.70	11.61	62.95	13.79	40.16	10.43	60.88			14.79	59.59
50	12.52	47.93	11.80	60.88	13.98	38.08	10.71	59.59			15.26	58.81
51	13.01	47.15	11.96	59.84	14.01	36.27	10.90	58.81			15.51	58.29
52	13.76	45.08	12.45	59.59	14.14	34.46	10.96	58.03			15.72	57.25
53	13.82	43.52	12.73	58.03	14.60	32.90	11.49	57.25			16.44	55.96
54	13.95	43.01	12.80	57.77	15.72	30.57	11.61	56.48			16.75	54.15
55	13.98	41.19	13.17	56.99	16.07	29.27	11.68	54.15			16.84	52.85
56	14.14	40.16	13.76	56.48	16.13	27.98	11.92	53.11			16.94	52.59
57	14.60	39.12	13.98	55.70	17.59	24.61	12.14	52.59			18.37	51.30
58	15.07	37.82	14.32	54.66	18.87	21.24	13.79	52.07			18.53	50.78
59	15.72	36.53	14.54	54.15	19.02	17.62	13.92	51.04			18.68	50.26
60	15.88	35.23	14.73	53.11	22.39	13.99	14.29	49.74			18.77	49.22
61	16.13	33.94	14.88	52.07	24.97	7.25	14.54	48.19			18.90	47.67
62	17.56	32.38	15.10	51.04			14.73	46.63			19.02	45.60
63	17.72	30.83	15.23	50.00			14.85	45.85			19.71	43.52
64	18.15	29.02	15.26	48.96			15.10	44.82			20.64	40.93
65	18.90	27.46	15.72	48.45			15.19	43.78			20.92	38.60
66	19.05	25.39	16.44	46.89			15.26	42.75			23.66	34.72
67	22.36	22.80	16.84	45.34			15.35	41.45				
68	22.45	19.95	17.44	44.04			16.41	40.16				
69	24.91	15.03	18.34	43.26			16.60	39.38				
70	25.10	10.36	18.68	42.49			17.47	38.34				
71			18.93	41.71			17.56	37.05				
72			19.02	40.93			19.46	35.23				
73			19.43	39.64			20.95	32.38				
74			19.71	38.86			22.60	28.50				
75			20.64	37.31			23.38	23.83				
76			20.89	36.01								
77			20.95	34.72								
78			22.57	33.16								
79			23.32	31.09								
80			23.66	29.27								

Supplementary Table S4 | Summary of statistical goodness-of-fit of Kaplan-Meier curves.

	**Exponential**	**Weibull**	**Log-logistic**	**Log-normal**	**Gompertz**
**Chemotherapy-PFS (PD-L1-positive)**					
parameter 1	0.15420560	0.05895870	1.59207100	1.57973100	0.11396230
parameter 2	-	0.38713920	-0.83187270	-0.24814730	0.06693550
AIC	478.71490000	447.94040000	427.28960000	433.72090000	471.03080000
BIC	481.92440000	454.35930000	433.70860000	440.13990000	477.44980000
**Chemotherapy-OS (PD-L1-positive)**					
parameter 1	0.05777860	0.01282380	2.56193300	2.52943000	0.03066500
parameter 2	-	0.41783890	-0.69616110	-0.12652130	0.05804070
AIC	478.20170000	448.73080000	455.28210000	459.48080000	454.07280000
BIC	481.41120000	455.14980000	461.70110000	465.89980000	460.49180000
**Serplulimab plus chemotherapy-PFS (PD-L1-positive)**					
parameter 1	0.11424750	0.06571900	1.80360200	1.79485500	0.10293160
parameter 2	-	0.20757360	-0.63588930	-0.07165520	0.01598830
AIC	1031.92900000	1013.07900000	981.96920000	984.95150000	1031.43900000
BIC	1035.83700000	1020.89500000	989.78540000	992.76760000	1039.25500000
**Serplulimab plus chemotherapy-OS (PD-L1-positive)**					
parameter 1	0.05008110	0.01236890	2.66730600	2.64454400	0.02813780
parameter 2	-	0.39475120	-0.64027740	-0.04762940	0.05482960
AIC	951.52080000	905.59380000	910.43480000	925.23200000	917.01660000
BIC	955.42880000	913.41000000	918.25090000	933.04820000	924.83280000
**Chemotherapy-PFS (PD-L1 1 ≤ CPS < 10)**					
parameter 1	0.17725530	0.04358060	1.52227600	1.49831700	0.09739300
parameter 2	-	0.55892000	-0.98024170	-0.40738260	0.15276290
AIC	258.23380000	224.05170000	216.44820000	218.11420000	241.44820000
BIC	260.87820000	229.34050000	221.73690000	223.40300000	246.73700000
**Chemotherapy-OS (PD-L1 1 ≤ CPS < 10)**					
parameter 1	0.07526070	0.00972660	2.39376000	2.34711400	0.03344240
parameter 2	-	0.55247840	-0.88564180	-0.30378400	0.08181930
AIC	259.77460000	226.13720000	233.71260000	236.28060000	232.19290000
BIC	262.41900000	231.42600000	239.00140000	241.56940000	237.48170000

**Serplulimab plus chemotherapy-PFS (PD-L1 1 ≤ CPS < 10)**					
parameter 1	0.12418240	0.07728060	1.71293700	1.70757500	0.12246710
parameter 2	-	0.18628100	-0.65409860	-0.07558770	0.00233380
AIC	583.00080000	575.36150000	547.82630000	552.77720000	584.97340000
BIC	586.32870000	582.01730000	554.48200000	559.43290000	591.62920000
**Serplulimab plus chemotherapy-OS (PD-L1 1 ≤ CPS < 10)**					
parameter 1	0.05949840	0.01853850	2.51308000	2.47058000	0.03496230
parameter 2	-	0.34514980	-0.60852820	0.00531460	0.05307060
AIC	553.03260000	531.70800000	540.21370000	553.55890000	534.68080000
BIC	556.36050000	538.36370000	546.86950000	560.21460000	541.33660000
**Chemotherapy-PFS (PD-L1 CPS ≥ 10)**					
parameter 1	0.14094150	0.06336350	1.65244500	1.62723600	0.10459860
parameter 2	-	0.32122150	-0.69583780	-0.12062290	0.06114160
AIC	214.60090000	207.14140000	204.01950000	206.05740000	212.95950000
BIC	216.97040000	211.88030000	208.75840000	210.79630000	217.69840000
**Chemotherapy-OS (PD-L1 CPS ≥ 10)**					
parameter 1	0.07034400	0.01804580	2.39867200	2.33479700	0.03386730
parameter 2	-	0.38773850	-0.65984120	-0.07991880	0.06666300
AIC	184.59760000	174.52870000	184.30080000	186.59720000	171.84140000
BIC	186.83170000	178.99690000	188.76900000	191.06540000	176.30960000
**Serplulimab plus chemotherapy-PFS (PD-L1 CPS ≥ 10)**					
parameter 1	0.08321670	0.04864210	2.08814100	2.08642500	0.07372920
parameter 2	-	0.19081790	-0.52911330	0.00366220	0.01641100
AIC	457.30040000	452.90290000	444.15080000	441.46520000	458.22790000
BIC	460.38800000	459.07810000	450.32600000	447.64040000	464.40310000
**Serplulimab plus chemotherapy-OS (PD-L1 CPS ≥ 10)**					
parameter 1	0.03923940	0.00630490	2.87705100	2.86606500	0.01930660
parameter 2	-	0.48103160	-0.68770110	-0.13080320	0.06314830
AIC	392.71600000	369.22820000	369.11910000	368.88580000	376.02730000
BIC	395.80360000	375.40340000	375.29430000	375.06100000	382.20250000
Optimal parameter distribution, AIC, Akaike information criterion; BIC, Bayesian Information Criterion; PFS, progression-free survival; OS, overall survival.					

Supplementary Table S5 | Long-term PFS data**.**

Month	PD-L1-positive		PD-L1 1 ≤ CPS < 10		PD-L1 CPS ≥ 10	
	Chemotherapy	Serplulimab	Chemotherapy	Serplulimab	Chemotherapy	Serplulimab
0	100.00%	100.00%	100.00%	100.00%	100.00%	100.00%
1	94.27%	96.79%	98.30%	96.42%	96.49%	98.12%
2	84.90%	89.06%	90.11%	87.67%	87.26%	91.75%
3	74.28%	79.11%	75.57%	76.52%	75.23%	83.75%
4	63.50%	68.74%	58.96%	65.21%	63.04%	75.73%
5	53.21%	59.07%	44.22%	54.96%	52.15%	68.27%
6	43.81%	50.56%	32.78%	46.22%	43.06%	61.55%
7	35.50%	43.32%	24.43%	38.98%	35.70%	55.57%
8	28.35%	37.26%	18.47%	33.07%	29.81%	50.28%
9	22.33%	32.23%	14.20%	28.26%	25.11%	45.60%
10	17.36%	28.04%	11.11%	24.34%	21.35%	41.47%
11	13.33%	24.56%	8.84%	21.12%	18.32%	37.82%
12	10.12%	21.64%	7.14%	18.47%	15.85%	34.57%
13	7.60%	19.19%	5.85%	16.26%	13.82%	31.68%
14	5.65%	17.11%	4.85%	14.41%	12.15%	29.09%
15	4.15%	15.34%	4.07%	12.85%	10.75%	26.78%
16	3.02%	13.82%	3.45%	11.53%	9.57%	24.71%
17	2.18%	12.51%	2.95%	10.39%	8.57%	22.84%
18	1.56%	11.38%	2.54%	9.41%	7.71%	21.16%
19	1.10%	10.39%	2.21%	8.56%	6.97%	19.63%
20	0.77%	9.52%	1.93%	7.82%	6.33%	18.25%
21	0.54%	8.76%	1.70%	7.17%	5.78%	16.99%
22	0.37%	8.08%	1.51%	6.59%	5.29%	15.84%
23	0.26%	7.48%	1.34%	6.09%	4.86%	14.80%
24	0.17%	6.94%	1.20%	5.64%	4.48%	13.84%
25	0.12%	6.46%	1.08%	5.23%	4.14%	12.96%
26	0.08%	6.03%	0.97%	4.87%	3.84%	12.15%
27	0.05%	5.63%	0.88%	4.55%	3.57%	11.41%
28	0.03%	5.28%	0.80%	4.25%	3.33%	10.73%
29	0.02%	4.96%	0.73%	3.98%	3.11%	10.09%
30	0.01%	4.66%	0.66%	3.74%	2.91%	9.51%
31	0.01%	4.40%	0.61%	3.52%	2.73%	8.97%
32	0.01%	4.15%	0.56%	3.32%	2.57%	8.47%
33	0.00%	3.93%	0.52%	3.14%	2.42%	8.00%
34	0.00%	3.72%	0.48%	2.97%	2.28%	7.57%
35	0.00%	3.53%	0.44%	2.81%	2.15%	7.17%
36	0.00%	3.35%	0.41%	2.67%	2.04%	6.79%
37	0.00%	3.19%	0.38%	2.53%	1.93%	6.44%
38	0.00%	3.04%	0.35%	2.41%	1.83%	6.11%

39	0.00%	2.89%	0.33%	2.29%	1.74%	5.80%
40	0.00%	2.76%	0.31%	2.19%	1.66%	5.52%
41	0.00%	2.64%	0.29%	2.09%	1.58%	5.25%
42	0.00%	2.53%	0.27%	1.99%	1.50%	5.00%
43	0.00%	2.42%	0.26%	1.91%	1.44%	4.76%
44	0.00%	2.32%	0.24%	1.83%	1.37%	4.54%
45	0.00%	2.22%	0.23%	1.75%	1.31%	4.33%
46	0.00%	2.14%	0.21%	1.68%	1.26%	4.13%
47	0.00%	2.05%	0.20%	1.61%	1.20%	3.94%
48	0.00%	1.97%	0.19%	1.55%	1.15%	3.77%
49	0.00%	1.90%	0.18%	1.49%	1.11%	3.60%
50	0.00%	1.83%	0.17%	1.43%	1.07%	3.45%
51	0.00%	1.76%	0.16%	1.38%	1.02%	3.30%
52	0.00%	1.70%	0.15%	1.33%	0.99%	3.16%
53	0.00%	1.64%	0.15%	1.28%	0.95%	3.03%
54	0.00%	1.59%	0.14%	1.24%	0.91%	2.90%
55	0.00%	1.53%	0.13%	1.20%	0.88%	2.78%
56	0.00%	1.48%	0.13%	1.16%	0.85%	2.67%
57	0.00%	1.43%	0.12%	1.12%	0.82%	2.56%
58	0.00%	1.39%	0.12%	1.08%	0.79%	2.46%
59	0.00%	1.35%	0.11%	1.05%	0.77%	2.36%
60	0.00%	1.30%	0.11%	1.01%	0.74%	2.27%
61	0.00%	1.26%	0.10%	0.98%	0.72%	2.18%
62	0.00%	1.23%	0.10%	0.95%	0.69%	2.10%
63	0.00%	1.19%	0.09%	0.92%	0.67%	2.02%
64	0.00%	1.16%	0.09%	0.90%	0.65%	1.95%
65	0.00%	1.12%	0.09%	0.87%	0.63%	1.87%
66	0.00%	1.09%	0.08%	0.85%	0.61%	1.81%
67	0.00%	1.06%	0.08%	0.82%	0.60%	1.74%
68	0.00%	1.03%	0.08%	0.80%	0.58%	1.68%
69	0.00%	1.00%	0.07%	0.78%	0.56%	1.62%
70	0.00%	0.98%	0.07%	0.76%	0.55%	1.56%
71	0.00%	0.95%	0.07%	0.74%	0.53%	1.51%
72	0.00%	0.93%	0.06%	0.72%	0.52%	1.45%
73	0.00%	0.90%	0.06%	0.70%	0.50%	1.40%
74	0.00%	0.88%	0.06%	0.68%	0.49%	1.36%
75	0.00%	0.86%	0.06%	0.66%	0.48%	1.31%
76	0.00%	0.84%	0.06%	0.65%	0.46%	1.27%
77	0.00%	0.82%	0.05%	0.63%	0.45%	1.23%
78	0.00%	0.80%	0.05%	0.62%	0.44%	1.18%
79	0.00%	0.78%	0.05%	0.60%	0.43%	1.15%
80	0.00%	0.76%	0.05%	0.59%	0.42%	1.11%
81	0.00%	0.74%	0.05%	0.57%	0.41%	1.07%

82	0.00%	0.73%	0.05%	0.56%	0.40%	1.04%
83	0.00%	0.71%	0.04%	0.55%	0.39%	1.01%
84	0.00%	0.70%	0.04%	0.53%	0.38%	0.98%
85	0.00%	0.68%	0.04%	0.52%	0.37%	0.94%
86	0.00%	0.67%	0.04%	0.51%	0.36%	0.92%
87	0.00%	0.65%	0.04%	0.50%	0.35%	0.89%
88	0.00%	0.64%	0.04%	0.49%	0.35%	0.86%
89	0.00%	0.62%	0.04%	0.48%	0.34%	0.83%
90	0.00%	0.61%	0.04%	0.47%	0.33%	0.81%
91	0.00%	0.60%	0.03%	0.46%	0.32%	0.79%
92	0.00%	0.59%	0.03%	0.45%	0.32%	0.76%
93	0.00%	0.57%	0.03%	0.44%	0.31%	0.74%
94	0.00%	0.56%	0.03%	0.43%	0.30%	0.72%
95	0.00%	0.55%	0.03%	0.42%	0.30%	0.70%
96	0.00%	0.54%	0.03%	0.41%	0.29%	0.68%
97	0.00%	0.53%	0.03%	0.41%	0.28%	0.66%
98	0.00%	0.52%	0.03%	0.40%	0.28%	0.64%
99	0.00%	0.51%	0.03%	0.39%	0.27%	0.62%
100	0.00%	0.50%	0.03%	0.38%	0.27%	0.60%
101	0.00%	0.49%	0.03%	0.38%	0.26%	0.59%
102	0.00%	0.48%	0.03%	0.37%	0.26%	0.57%
103	0.00%	0.47%	0.02%	0.36%	0.25%	0.56%
104	0.00%	0.47%	0.02%	0.35%	0.25%	0.54%
105	0.00%	0.46%	0.02%	0.35%	0.24%	0.53%
106	0.00%	0.45%	0.02%	0.34%	0.24%	0.51%
107	0.00%	0.44%	0.02%	0.34%	0.23%	0.50%
108	0.00%	0.43%	0.02%	0.33%	0.23%	0.49%
109	0.00%	0.43%	0.02%	0.32%	0.23%	0.47%
110	0.00%	0.42%	0.02%	0.32%	0.22%	0.46%
111	0.00%	0.41%	0.02%	0.31%	0.22%	0.45%
112	0.00%	0.40%	0.02%	0.31%	0.21%	0.44%
113	0.00%	0.40%	0.02%	0.30%	0.21%	0.43%
114	0.00%	0.39%	0.02%	0.30%	0.21%	0.41%
115	0.00%	0.39%	0.02%	0.29%	0.20%	0.40%
116	0.00%	0.38%	0.02%	0.29%	0.20%	0.39%
117	0.00%	0.37%	0.02%	0.28%	0.20%	0.38%
118	0.00%	0.37%	0.02%	0.28%	0.19%	0.37%
119	0.00%	0.36%	0.02%	0.27%	0.19%	0.36%
120	0.00%	0.36%	0.02%	0.27%	0.19%	0.36%

Supplementary Table S6 | Long-term OS data.

Month	PD-L1-positive		PD-L1 1 ≤ CPS < 10		PD-L1 CPS ≥ 10	
	Chemotherapy	Serplulimab	Chemotherapy	Serplulimab	Chemotherapy	Serplulimab
0	100.00%	100.00%	100.00%	100.00%	100.00%	100.00%
1	98.73%	99.37%	99.70%	99.02%	99.04%	99.95%
2	96.39%	97.69%	98.41%	96.59%	96.44%	99.34%
3	93.42%	95.15%	95.85%	93.08%	92.52%	97.80%
4	90.01%	91.91%	92.00%	88.80%	87.64%	95.42%
5	86.26%	88.15%	87.01%	84.03%	82.15%	92.40%
6	82.29%	84.04%	81.15%	79.01%	76.39%	88.96%
7	78.17%	79.71%	74.76%	73.93%	70.60%	85.29%
8	73.96%	75.31%	68.18%	68.93%	64.97%	81.50%
9	69.72%	70.93%	61.69%	64.12%	59.62%	77.71%
10	65.49%	66.64%	55.50%	59.55%	54.63%	73.96%
11	61.31%	62.51%	49.75%	55.27%	50.04%	70.32%
12	57.21%	58.57%	44.50%	51.29%	45.84%	66.80%
13	53.23%	54.84%	39.77%	47.62%	42.03%	63.43%
14	49.38%	51.34%	35.55%	44.24%	38.58%	60.21%
15	45.67%	48.07%	31.82%	41.14%	35.47%	57.15%
16	42.13%	45.02%	28.53%	38.30%	32.67%	54.24%
17	38.76%	42.20%	25.63%	35.70%	30.14%	51.49%
18	35.57%	39.58%	23.08%	33.33%	27.86%	48.89%
19	32.56%	37.15%	20.83%	31.16%	25.81%	46.44%
20	29.73%	34.91%	18.85%	29.17%	23.96%	44.13%
21	27.08%	32.84%	17.11%	27.36%	22.28%	41.94%
22	24.61%	30.92%	15.57%	25.69%	20.76%	39.88%
23	22.32%	29.15%	14.21%	24.16%	19.38%	37.94%
24	20.19%	27.51%	12.99%	22.76%	18.13%	36.11%
25	18.23%	25.99%	11.91%	21.47%	16.99%	34.38%
26	16.42%	24.59%	10.95%	20.28%	15.94%	32.75%
27	14.76%	23.28%	10.09%	19.18%	14.99%	31.21%
28	13.24%	22.07%	9.32%	18.16%	14.11%	29.76%
29	11.85%	20.95%	8.62%	17.22%	13.31%	28.39%
30	10.59%	19.91%	8.00%	16.35%	12.57%	27.10%
31	9.44%	18.93%	7.43%	15.55%	11.89%	25.87%
32	8.40%	18.03%	6.92%	14.80%	11.26%	24.72%
33	7.46%	17.18%	6.45%	14.10%	10.68%	23.62%
34	6.62%	16.39%	6.03%	13.45%	10.14%	22.59%
35	5.86%	15.65%	5.64%	12.84%	9.64%	21.60%
36	5.17%	14.96%	5.29%	12.27%	9.18%	20.68%
37	4.56%	14.31%	4.97%	11.74%	8.75%	19.80%
38	4.02%	13.70%	4.67%	11.24%	8.34%	18.96%

39	3.53%	13.13%	4.40%	10.77%	7.97%	18.17%
40	3.09%	12.59%	4.15%	10.33%	7.61%	17.42%
41	2.71%	12.08%	3.92%	9.92%	7.29%	16.70%
42	2.37%	11.60%	3.70%	9.53%	6.98%	16.03%
43	2.07%	11.15%	3.50%	9.16%	6.69%	15.38%
44	1.80%	10.73%	3.32%	8.82%	6.41%	14.77%
45	1.57%	10.33%	3.15%	8.49%	6.16%	14.19%
46	1.36%	9.95%	2.99%	8.18%	5.92%	13.63%
47	1.18%	9.59%	2.84%	7.89%	5.69%	13.10%
48	1.02%	9.25%	2.71%	7.61%	5.48%	12.60%
49	0.88%	8.92%	2.58%	7.35%	5.27%	12.12%
50	0.76%	8.62%	2.46%	7.10%	5.08%	11.66%
51	0.66%	8.33%	2.35%	6.87%	4.90%	11.22%
52	0.56%	8.05%	2.24%	6.64%	4.73%	10.81%
53	0.48%	7.79%	2.14%	6.43%	4.56%	10.41%
54	0.42%	7.54%	2.05%	6.23%	4.41%	10.03%
55	0.36%	7.30%	1.96%	6.03%	4.26%	9.67%
56	0.30%	7.07%	1.88%	5.85%	4.12%	9.32%
57	0.26%	6.85%	1.80%	5.67%	3.99%	8.99%
58	0.22%	6.64%	1.73%	5.50%	3.86%	8.67%
59	0.19%	6.45%	1.66%	5.34%	3.74%	8.37%
60	0.16%	6.26%	1.59%	5.19%	3.63%	8.08%
61	0.14%	6.07%	1.53%	5.04%	3.52%	7.80%
62	0.12%	5.90%	1.47%	4.90%	3.41%	7.53%
63	0.10%	5.73%	1.42%	4.76%	3.31%	7.28%
64	0.08%	5.57%	1.37%	4.63%	3.21%	7.03%
65	0.07%	5.42%	1.32%	4.51%	3.12%	6.80%
66	0.06%	5.28%	1.27%	4.39%	3.03%	6.57%
67	0.05%	5.13%	1.22%	4.27%	2.95%	6.35%
68	0.04%	5.00%	1.18%	4.16%	2.87%	6.15%
69	0.04%	4.87%	1.14%	4.06%	2.79%	5.95%
70	0.03%	4.74%	1.10%	3.96%	2.72%	5.76%
71	0.02%	4.62%	1.07%	3.86%	2.64%	5.57%
72	0.02%	4.51%	1.03%	3.77%	2.58%	5.39%
73	0.02%	4.40%	1.00%	3.67%	2.51%	5.22%
74	0.01%	4.29%	0.96%	3.59%	2.45%	5.06%
75	0.01%	4.19%	0.93%	3.50%	2.38%	4.90%
76	0.01%	4.09%	0.90%	3.42%	2.33%	4.75%
77	0.01%	3.99%	0.88%	3.34%	2.27%	4.61%
78	0.01%	3.90%	0.85%	3.27%	2.21%	4.47%
79	0.01%	3.81%	0.82%	3.19%	2.16%	4.33%
80	0.00%	3.72%	0.80%	3.12%	2.11%	4.20%
81	0.00%	3.64%	0.78%	3.05%	2.06%	4.08%

82	0.00%	3.56%	0.75%	2.99%	2.01%	3.95%
83	0.00%	3.48%	0.73%	2.92%	1.97%	3.84%
84	0.00%	3.40%	0.71%	2.86%	1.92%	3.73%
85	0.00%	3.33%	0.69%	2.80%	1.88%	3.62%
86	0.00%	3.26%	0.67%	2.75%	1.84%	3.51%
87	0.00%	3.19%	0.65%	2.69%	1.80%	3.41%
88	0.00%	3.13%	0.64%	2.63%	1.76%	3.31%
89	0.00%	3.06%	0.62%	2.58%	1.72%	3.22%
90	0.00%	3.00%	0.60%	2.53%	1.69%	3.13%
91	0.00%	2.94%	0.59%	2.48%	1.65%	3.04%
92	0.00%	2.88%	0.57%	2.43%	1.62%	2.96%
93	0.00%	2.82%	0.56%	2.39%	1.59%	2.88%
94	0.00%	2.77%	0.54%	2.34%	1.55%	2.80%
95	0.00%	2.72%	0.53%	2.30%	1.52%	2.72%
96	0.00%	2.66%	0.52%	2.25%	1.49%	2.65%
97	0.00%	2.61%	0.50%	2.21%	1.46%	2.57%
98	0.00%	2.56%	0.49%	2.17%	1.44%	2.51%
99	0.00%	2.52%	0.48%	2.13%	1.41%	2.44%
100	0.00%	2.47%	0.47%	2.09%	1.38%	2.37%
101	0.00%	2.42%	0.46%	2.06%	1.36%	2.31%
102	0.00%	2.38%	0.45%	2.02%	1.33%	2.25%
103	0.00%	2.34%	0.43%	1.99%	1.31%	2.19%
104	0.00%	2.30%	0.42%	1.95%	1.28%	2.13%
105	0.00%	2.26%	0.42%	1.92%	1.26%	2.08%
106	0.00%	2.22%	0.41%	1.89%	1.24%	2.03%
107	0.00%	2.18%	0.40%	1.85%	1.21%	1.97%
108	0.00%	2.14%	0.39%	1.82%	1.19%	1.92%
109	0.00%	2.10%	0.38%	1.79%	1.17%	1.87%
110	0.00%	2.07%	0.37%	1.76%	1.15%	1.83%
111	0.00%	2.04%	0.36%	1.74%	1.13%	1.78%
112	0.00%	2.00%	0.36%	1.71%	1.11%	1.74%
113	0.00%	1.97%	0.35%	1.68%	1.09%	1.69%
114	0.00%	1.94%	0.34%	1.65%	1.08%	1.65%
115	0.00%	1.91%	0.33%	1.63%	1.06%	1.61%
116	0.00%	1.87%	0.33%	1.60%	1.04%	1.57%
117	0.00%	1.85%	0.32%	1.58%	1.02%	1.53%
118	0.00%	1.82%	0.31%	1.55%	1.01%	1.50%
119	0.00%	1.79%	0.31%	1.53%	0.99%	1.46%
120	0.00%	1.76%	0.30%	1.51%	0.97%	1.43%

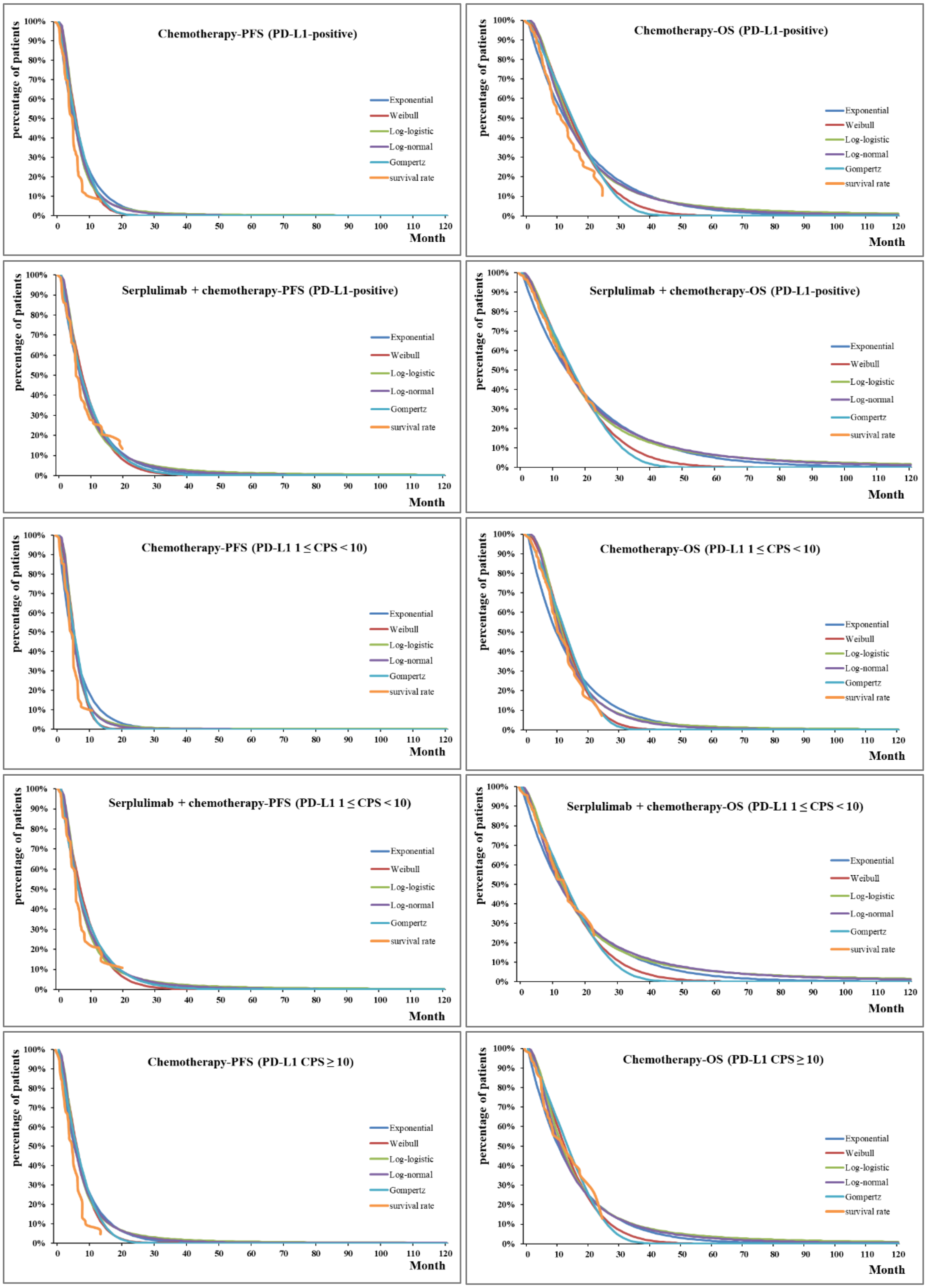
Chemotherapy-PFS (PD-L1-positive)
Chemotherapy-OS (PD-L1-positive)
Serplulimab + chemotherapy-PFS (PD-L1-positive)
Serplulimab + chemotherapy-OS (PD-L1-positive)
Chemotherapy-PFS (PD-L1 1 ≤ CPS < 10)
Chemotherapy-OS (PD-L1 1 ≤ CPS < 10)
Serplulimab + chemotherapy-PFS (PD-L1 1 ≤ CPS < 10)
Serplulimab + chemotherapy-OS (PD-L1 1 ≤ CPS < 10)
Chemotherapy-PFS (PD-L1 CPS ≥ 10)
Chemotherapy-OS (PD-L1 CPS ≥ 10)
percentage of patients
Month
Exponential
Weibull
Log-logistic
Log-normal
Gompertz
survival rate

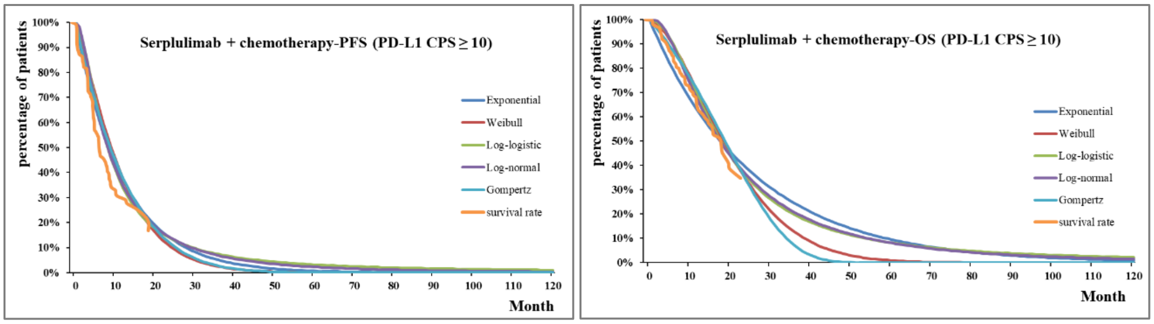

Supplementary Figure S1 | The reconstructed Kaplan-Meier PFS and OS curves.

Supplementary Table S7 | Scenario analysis results of various time horizons.

Parameters	All patients		Patients with 1 ≤ CPS < 10		Patients with CPS ≥ 10	
	C	S+C	C	S+C	C	S+C
**Time horizon = 2 years**						
**OS**						
Total cost ($)	4,644.47	33,238.96	4,126.24	30,790.98	4,466.25	41,634.74
QALYs	0.76	0.82	0.67	0.75	0.70	0.92
ICER ($/QALY)		513,489.22		341,047.31		165,144.80
**Only PFS**						
Total cost ($)	2,785.92	32,355.96	2,560.08	29,993.60	3,177.43	40,834.60
QALYs	0.38	0.50	0.35	0.46	0.43	0.63
ICER ($/QALY)		247,761.87		243,613.73		186,909.92
**Time horizon = 5 years**						
**OS**						
Total cost ($)	4,998.03	37,189.63	4,501.02	33,950.46	5,187.40	49,112.43
QALYs	0.83	1.03	0.74	0.92	0.83	1.22
ICER ($/QALY)		164,908.54		164,525.70		112,793.22
**Only PFS**						
Total cost ($)	2,787.42	35,875.82	2,612.48	32,795.23	3,431.60	47,806.02
QALYs	0.38	0.56	0.36	0.51	0.47	0.75
ICER ($/QALY)		188,129.85		200,364.09		158,901.79
**Time horizon = 8 years**						
**OS**						
Total cost ($)	4,999.71	38,161.25	4,562.88	34,709.80	5,362.40	50,542.96
QALYs	0.83	1.09	0.76	0.97	0.86	1.28
ICER ($/QALY)		131,997.65		141,047.82		106,521.71
**Only PFS**						
Total cost ($)	2,787.42	36,729.31	2,619.40	33,453.75	3,487.57	49,109.58
QALYs	0.38	0.57	0.36	0.52	0.47	0.77
ICER ($/QALY)		178,982.50		192,338.34		155,890.96

S+C, Serplulimab plus chemotherapy; C, chemotherapy; QALYs, quality-adjusted life years, ICER, incremental cost-effectiveness ratios.

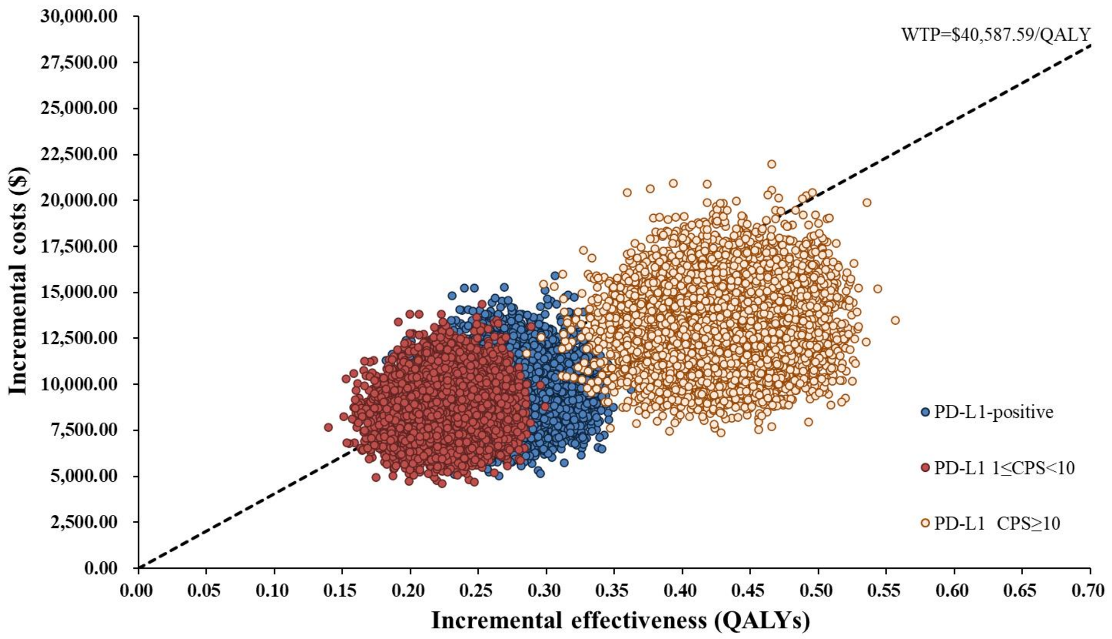

Supplementary Figure S2 | Scatter Plot of the ICER between serplulimab plus chemotherapy and chemotherapy (serplulimab at 30% of current price). WTP, willingness-to-pay; QALY, quality-adjusted life years.

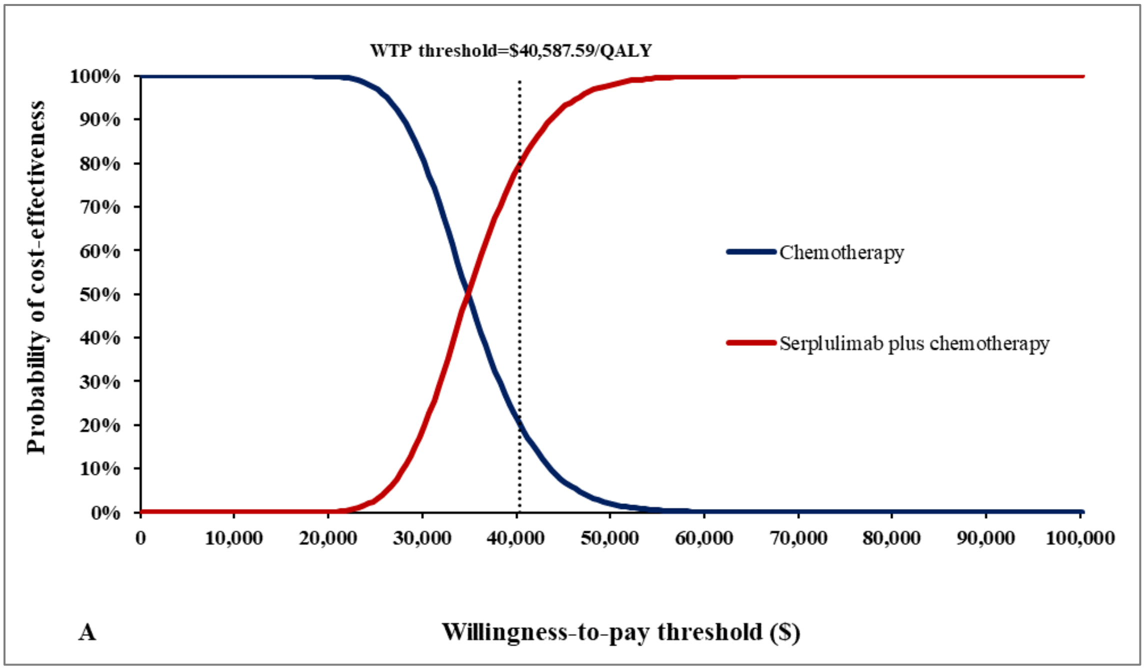
WTP threshold=$40,587.59/QALY
100%
90%
80%
70%
60%
50%
40%
30%
20%
10%
0%
Probability of cost-effectiveness
0
10,000
20,000
30,000
40,000
50,000
60,000
70,000
80,000
90,000
100,000
Chemotherapy
Serplulimab plus chemotherapy
A
Willingness-to-pay threshold ($)

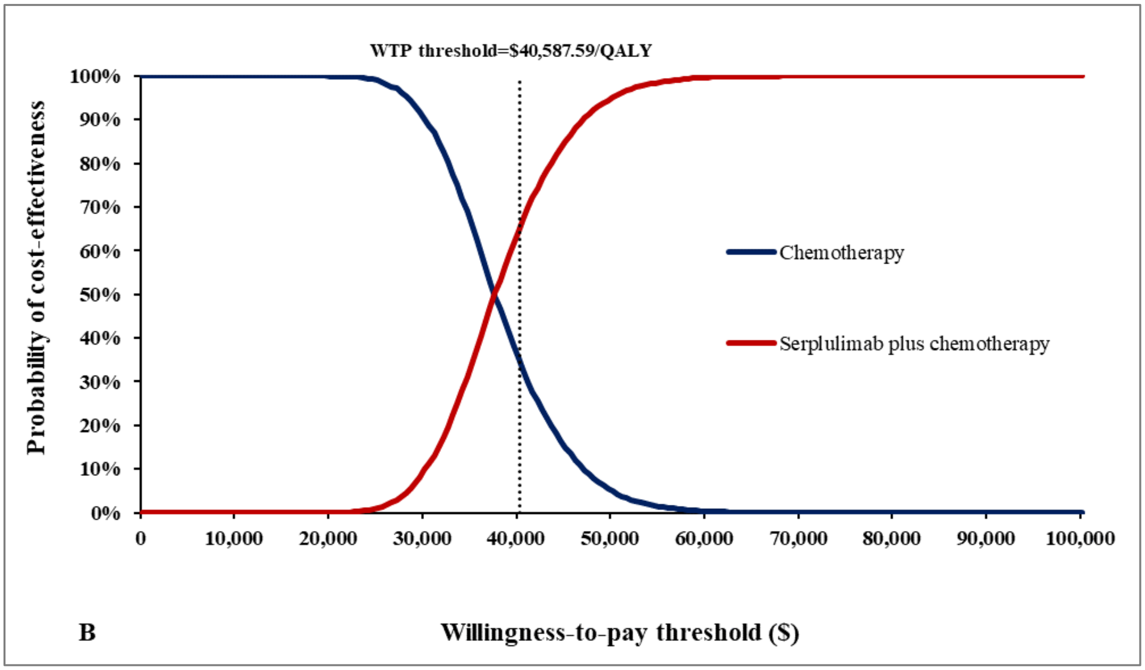
WTP threshold=$40,587.59/QALY
100%
90%
80%
70%
60%
50%
40%
30%
20%
10%
0%
Probability of cost-effectiveness
0
10,000
20,000
30,000
40,000
50,000
60,000
70,000
80,000
90,000
100,000
Chemotherapy
Serplulimab plus chemotherapy
B
Willingness-to-pay threshold ($)

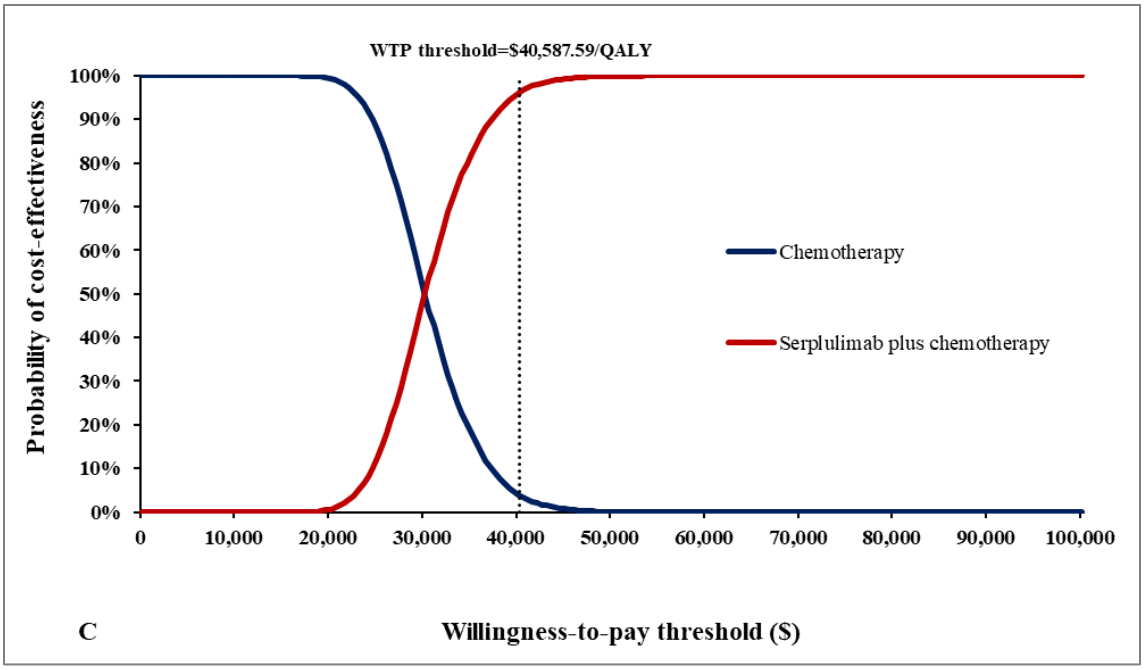

Supplementary Figure S3 | Cost-effectiveness acceptability curves for serplulimab plus chemotherapy versus chemotherapy (serplulimab at 30% of current price). (A: all PD-L1-positive advanced esophageal squamous cell carcinoma patients; B: Patients with PD-L1 $1 \leq$ CPS < 10; C: Patients with PD-L1 CPS ≥ 10) WTP, willingness-to-pay.
